# Advancements in Targeted Quantum Dots Structures for Enhanced Cancer Treatment

**DOI:** 10.3390/pharmaceutics17111396

**Published:** 2025-10-28

**Authors:** Nutan Shukla, Carol Y. Cárdenas, Aayushi Chanderiya, Oleg E. Polozhentsev, Ratnesh Das, Supriya Vyas, Elizaveta Mukhanova, Alexander Soldatov, Sabrina Belbekhouche

**Affiliations:** 1 The Smart Materials Research Institute, Southern Federal University, Rostov-on-Don 344090, Russia; carolcardenas972@gmail.com (C.Y.C.); olegpolozhentsev@mail.ru (O.E.P.); kand@sfedu.ru (E.M.); soldatov@sfedu.ru (A.S.); 2 Department of Chemistry, Dr. Harisingh Gour University, Sagar 470003, MP, India; chanderiyaayushi@gmail.com (A.C.); rdas@dhsgsu.edu.in (R.D.); 3 Faculty of Science, Shri Vaishnav Vidyapeeth Vishwavidyalaya, Indore 453111, MP, India; vyas@gmail.com; 4 Institut Chimie et Matériaux Paris Est, CNRS, Université Paris Est Creteil, UMR 7182, 2 Rue Henri Dunant, 94320 Thiais, France

**Keywords:** targeted quantum dots (TQDs), cancer therapy, combinational therapy, imaging, theranostics, functionalization

## Abstract

Quantum dots (QDs) have emerged as promising nanomaterials in cancer therapeutics owing to their tunable optical properties, versatile surface functionalization, and potential for simultaneous imaging and drug delivery. This review focuses on targeted quantum dots (TQDs), highlighting their role in overcoming the limitations of passive drug delivery strategies, such as poor specificity, high systemic toxicity, and limited therapeutic efficacy. We begin by outlining the fundamentals of QDs, including their types, heterostructures, and biomedical formulations. Recent advances in tailoring QD physicochemical properties to the cancer microenvironment are discussed, with emphasis on routes of administration and targeting strategies. The review critically examines different molecular targeting approaches—such as folate receptors, transferrin receptors, aptamers, antibodies, peptides, and hyaluronic acid—used to enhance therapeutic precision. Furthermore, we summarize progress in TQD-based combination therapies, including chemotherapy–photodynamic therapy, photothermal therapy, radiotherapy, and multimodal platforms that integrate therapy with imaging. Special attention is given to the role of QDs in theranostic, hydrogels, nanocomposites, and hybrid systems that enable controlled drug release and real-time monitoring. Despite significant advancements, challenges remain regarding biocompatibility, safety, and regulatory approval. Overall, this review provides an integrative perspective on the design, functionalization, and biomedical applications of TQDs, underscoring their potential to improve cancer treatment outcomes through enhanced specificity, reduced side effects, and multifunctional theranostic capabilities. Highlight of novelty: This review uniquely emphasizes the latest advances in targeted quantum dots (TQDs), particularly in surface functionalization, hybrid nanostructures, biodistribution, and multimodal theranostic applications, providing an updated perspective that extends beyond conventional QD-based cancer therapies.

## 1. Introduction

Nanotechnology has revolutionized cancer research by enabling the design of nanoscale platforms for targeted drug delivery, diagnostic imaging, and theranostics [1,2]. Among various nanomaterials, quantum dots (QDs) have emerged as one of the most promising candidates due to their unique optical and electronic properties, including size-tunable fluorescence, exceptional photostability, and high quantum yield [3,4]. These attributes make QDs highly suitable for biomedical applications such as tumor imaging, biosensing, and drug delivery [5,6]. However, the translation of traditional QDs into clinical practice remains limited owing to challenges related to biocompatibility, toxicity, biodistribution, and in vivo stability [7,8,9].

Recent advances in nanomedicine have introduced targeted quantum dots (TQDs)—engineered QDs functionalized with surface ligands, peptides, antibodies, or aptamers to achieve selective interactions with cancer cells [10,11]. Unlike conventional QDs that rely on passive targeting mechanisms, TQDs enable active targeting, enhancing therapeutic precision, minimizing off-target effects, and facilitating real-time monitoring of therapeutic responses [12,13]. While several reviews have broadly summarized the biomedical applications of QDs in cancer nanotechnology [14,15], a comprehensive and critical evaluation of recent progress in TQD design, hybrid nanostructures, surface functionalization strategies, and multimodal theranostic applications remains scarce [13,16].

The present review seeks to address this gap by critically examining emerging trends in the design, functionalization, and application of TQDs for cancer therapy. Specifically, it highlights four underexplored dimensions: advanced surface functionalization strategies that enhance stability, targeting efficiency, and biocompatibility [13,17]; comprehensive insights into biocompatibility and biodistribution, which are crucial for ensuring safe in vivo performance [5]; the development of hybrid and multifunctional nanostructures that seamlessly integrate imaging and therapeutic functionalities [18,19]; and the evolution of multimodal theranostic platforms that illustrate the transformation of TQDs from passive carriers into precision-guided nanotherapeutics [20]. By integrating these perspectives, this review not only extends the scope of existing literature [20,21] but also provides a contemporary framework for understanding how next-generation TQDs are advancing toward clinical translation in precision cancer nanotheranostics. Typically ranging from 1 to 10 nanometers in diameter, QDs are semiconductor nanocrystals composed of elements from groups II–VI (e.g., CdSe, CdTe, CdS, ZnSe, ZnTe, ZnS), III–V (e.g., InAs, InP), I–III–VI_2_ (e.g., CuInS_2_, AgInS_2_), IV–VI (e.g., PbS, PbSe, PbTe), or group IV elements (e.g., C, Si, Ge). Their remarkable optical behavior originates from quantum confinement effects, which occur when particle dimensions approach the exciton Bohr radius. This confinement leads to discrete energy levels analogous to atomic orbitals, causing QDs to behave like “artificial atoms” [22]. The emission wavelength of QDs is directly correlated with their size: smaller QDs emit blue-shifted light (shorter wavelength), while larger QDs exhibit red-shifted emission [1]. This size-dependent tunability underpins their utility across electronics, optoelectronics, and particularly biomedical imaging [23].

In cancer nanomedicine, QDs have been utilized in fluorescence imaging to visualize cellular and subcellular structures with high spatial resolution [5]. Their narrow emission spectra facilitate multiplexed imaging, allowing simultaneous tracking of multiple biomarkers [3]. When conjugated with targeting ligands, QDs can selectively bind to specific cancer cell receptors, enabling targeted drug delivery and biosensing at molecular levels [11,24]. Moreover, QDs exhibit potential for photothermal and photodynamic therapy, where light-induced excitation generates localized heating or reactive oxygen species to selectively damage tumor cells [19]. Nevertheless, optimizing delivery mechanisms, biodistribution, tumor microenvironment interactions, and clearance pathways remains critical for improving the safety and efficacy of QD-based therapeutics [25,26].

This review thus offers a comprehensive insight into the evolution of tailored quantum dot systems in cancer treatment, emphasizing technological advancements that enhance therapeutic precision, biocompatibility, and efficacy [13,25]. It highlights recent innovations in surface modification, such as custom ligand engineering, enhanced drug encapsulation, and stimuli-responsive release systems, which collectively contribute to developing next-generation QD-based therapies [13,17]. These systems promise reduced systemic toxicity, improved selectivity, and personalized therapeutic outcomes [12]. Furthermore, the review discusses the integration of QDs into combination therapies and their potential for real-time monitoring of treatment efficacy, underscoring their growing relevance in precision oncology [27,28].

## 2. Fundamentals of Quantum Dots

### 2.1. Types of Quantum Dots

**Figure 1:**
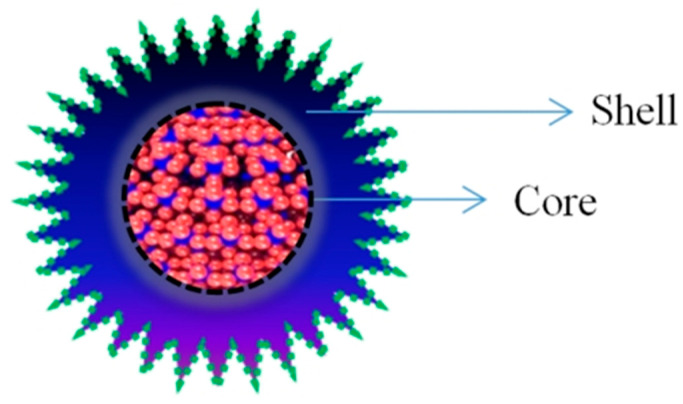
Representation of core–shell structure of QD’s.

Core and Core–Shell Quantum Dots: Quantum dots (QDs) can be broadly classified into core and core–shell structures. Core quantum dots are the simplest form, consisting of a single semiconductor core that defines their fundamental optical and electronic properties. Commonly used materials include cadmium selenide (CdSe), cadmium sulfide (CdS), and lead sulfide (PbS) [29]. In contrast, core–shell quantum dots comprise a semiconductor core encapsulated by a shell made of a different semiconductor material. The presence of the shell enhances the photoluminescence efficiency, chemical stability, and overall performance of the quantum dots. For instance, CdSe/CdS QDs are frequently employed in biomedical applications due to their higher quantum yield and reduced toxicity [14,30] (Figure 1).

Doped Quantum Dots: These QDs are intentionally doped with other elements to modify their electronic properties. This can enhance their functionality in specific applications, such as improving the sensitivity of biosensors [14].

Heterostructured Quantum Dots: These consist of different semiconductor materials combined in a single quantum dot, allowing for tailored electronic and optical properties. Heterostructures can facilitate multiple functionalities, such as simultaneous imaging and therapeutic applications [21].

### 2.2. Heterostructure of QDs for Biomedical Application

Researchers have combined QDs with other materials to create heterostructures, which show the versatility and potential of combining different materials to create multifunctional nanoplatform for a spectrum of biomedical uses, consisting of biosensing, drug delivery, phototherapy, and bio imaging. This has allowed researchers to improve the performance of QDs for numerous biomedical applications as shown in Table 1. These heterostructures take advantage of the materials’ synergistic qualities, like [31].

Graphene/MoS_2_: Graphene, a two-dimensional material with exceptional mechanical, electrical, and optical properties, has been combined with molybdenum disulfide (MoS_2_), another 2D material with strong light-matter interactions, to form heterostructures for developing biomedical devices [22].

CdS: Cadmium sulfide (CdS) QDs are widely used in photocatalysis and have been combined with other materials, such as ZnS, to form core/shell heterostructures. The CdS core provides suitable bandgap positions for redox reactions and photocatalyzed processes, while the ZnS shell improves stability and reduces charge recombination [23].

ZnSe-CdS: Zinc selenide (ZnSe)-cadmium sulfide (CdS) heterostructure has been developed to augment the catalytic function of CdS QDs. The combination of these materials reduces charge recombination and enhances the number of surface-active sites, leading to improved photo catalytic efficiency [32].

CuInS_2_-ZnS: Copper indium sulfide (CuInS_2_) QDs combined with a zinc sulfide (ZnS) shell have been used for the detection of human Interleukin 6, a biomarker for various diseases. These heterostructures are synthesized using aqueous methods to achieve high luminescence and biocompatibility [33].

AgInS_2_-ZnS: Silver indium sulfide (AgInS_2_) QDs with a ZnS shell have been developed for biological applications. The aqueous synthesis method used to prepare these heterostructures results in high luminescence and improved biocompatibility [34].

**Table 1:** Summarize instances of structural types and heterostructure, as well as materials employed to produce QD’s.

Type of Structure and Heterostructure	Material Used	Reference
Fish Scale-Derived Carbon Dots (FS-CDs)	fish scale	[35]
Phosphorus-doped CQDs (P-CQDs)	yeast cell walls	[36]
Fluorescent QD	based hydrogels	[37]
CuQDs	CuInS_2_	[38]
Multifunctional microspheres (MFM)	Fluorescent source (CdSe/ZnS quantum dots), silica nanoparticles	[39]
CdSe:ZnS QDs	CdSe core—ZnS shells	[40]
Fluorescent carbon quantum dots (CQDs)—FS-CDs	Aegle marmelos fruit extract	[41]

### 2.3. Quantum Dot-Based Formulation for Biomedical Applications

An effective quantum dot (QD) formulation should ensure biocompatibility, controlled biodistribution and clearance, selective targeting, and therapeutic efficacy. Minimizing toxicity is critical [24]. While cadmium-based QDs exhibit high quantum yields, their toxicity limits biomedical use; safer alternatives such as silicon- and carbon-based QDs offer favorable profiles without compromising performance. Understanding biodistribution and metabolic pathways is essential, as QDs often accumulate in the liver and kidneys, requiring optimized clearance mechanisms [25]. An ideal formulation should achieve rapid clearance from non-target issues while maintaining sufficient retention at the disease site [42].

Surface modification with ligands or antibodies enables selective recognition of tumor-specific receptors, improving targeting and reducing off-target effects [43]. Stimulus-responsive elements triggered by enzymatic activity, pH, or temperature within the tumor microenvironment can further enhance site-specific drug release [44]. Biocompatible coatings, such as polyethylene glycol, reduce immunogenicity, improve circulation, and prevent aggregation while aiding cellular uptake [45]. Additionally, QDs engineered to absorb near-infrared (NIR) light can generate localized heat or reactive oxygen species, enabling tumor ablation and imaging. Multifunctional formulations incorporating chemotherapeutics or immunotherapeutics within the QD matrix allow synergistic effects and enhanced efficacy [46,47].

Advancement of QD-based therapeutics depends on optimizing targeting, safety, and long-term efficacy. Addressing these aspects through innovative designs and pharmacokinetic studies is vital for successful clinical translation [48,49,50]. QDs also surpass conventional fluorophores in stability and luminescence, making them highly effective in bioimaging (Table 2). Their conjugation with biomolecules such as proteins and oligonucleotides facilitates cellular and molecular studies, while applications in ex vivo patient tissues improve diagnosis and drug development [51]. Core–shell QDs, with their superior optoelectronic and narrow emission properties, are promising for diagnostics, drug delivery, and theranostic. Recent advances in synthesis and bioconjugation have expanded their integration into hybrid nanomaterials with significant potential in disease management [51,52].

**Figure 2:**
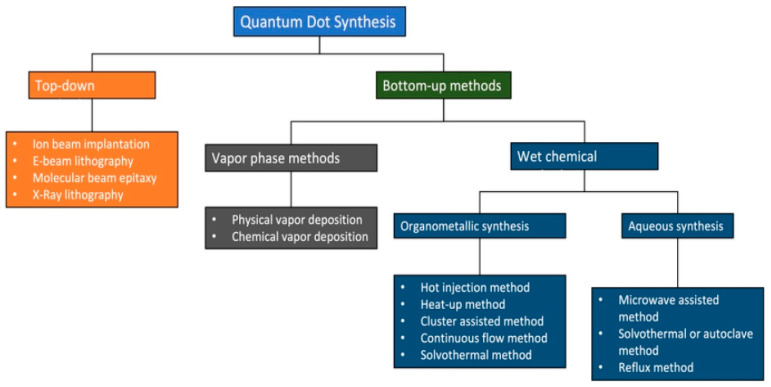
Flowchart of different routes of administration and endovascular targeting vs. extra vascular targeting. Reproduced, open access, license CC BY 4.0, from [65].

Advancement in synthesis techniques such as hydrothermal, solvothermal, microwave, aqueous phase synthesis, sol-gel method, polyol method, as is summarized in Figure 2, and Table 3, co-precipitation and microfluidics have made the synthesis of CSQDs possible in various ways [43]. The core-shell configuration of quantum dot, in which the inner core material is enclosed in an outer protective shell, is a standard design for biomedical applications; this structure allows you to customize QD properties such as luminescence and stability by varying the materials present in the inner core and outer shell. QD might likewise be conjugated with different kinds of molecules, in particular antibodies; to target specific cells or biomarkers [63]. Recent studies have highlighted the application of QD in a range of biomedical fields, including detection: Biomarkers of breast cancer, exosomes of cancerous origin, folate receptor-positive cancer cells. Potential of quantum dots in two-modal fluorescence technique and magnetic resonance imaging has also been investigated [55,64].

## 3. Quantum Dot-Based Cancer Care Drugs

### 3.1. Cancer Environment and Location

**Figure 3:**
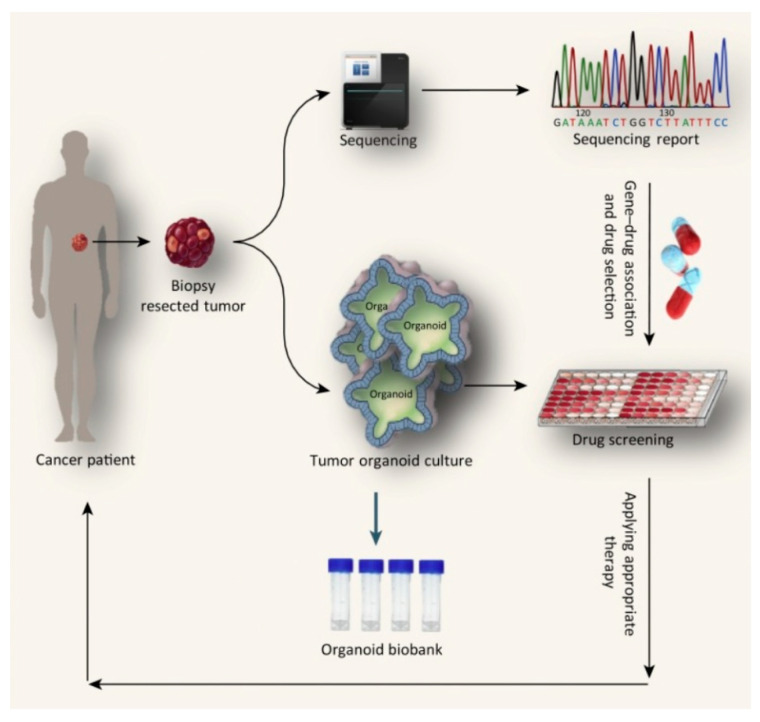
Schematic illustartion of Organoid-Based Personalized Cancer Therapy. Reprinted from: with permission from Elsevier [83].

Cancer treatment faces challenges due to the complexity of tumor biology, the tumor microenvironment (TME), and tumor location, along with the limitations of conventional therapies such as surgery, chemotherapy, immunotherapy, and radiation [43]. Tumor organoids derived from different locations provide promising models for personalized therapy, drug screening, and outcome prediction (Figure 3) [76]. The TME comprises cancer cells, stromal cells, extracellular matrix (ECM), cytokines, and vasculature, all of which support tumor progression [77,78]. Stromal components, including immune cells, fibroblasts, and endothelial cells, play critical roles in tumor development and immune modulation [22,23,32]. The ECM provides structural and biochemical support [61,79], while tumor angiogenesis sustains growth but often results in abnormal vasculature, hypoxia, and necrosis [80]. Cancer cell location strongly affects diagnosis, treatment planning, and outcomes. Tumor spread involves primary sites, circulating tumor cells (CTCs), and metastases [81,82].

CTCs enable distant colonization, often preceded by lymph node invasion. Within tumors, heterogeneous regions arise, including well-perfused zones, necrotic areas, and disordered vasculatures, contributing to therapeutic variability. The TME exhibits unique physicochemical features that influence therapy response. Hypoxia induces angiogenesis and metabolic shifts [84]; acidosis (pH 6.0–7.0 extracellular vs. ~7.0–7.2 intracellular) impairs immune function [43,55,85]; and increased stiffness and pressure hinder drug delivery [53,54]. Tumor–stroma interactions further enhance survival and heterogeneity [86,87], complicating therapy. Resistance arises via genetic mutations, metabolic adaptation, DNA repair, and microenvironmental protection. Cancer stem cells (CSCs) also drive initiation, relapse, and resistance, yet remain difficult to target [56,58,62]. Routes of drug delivery depend on tumor location and disease stage. Systemic approaches, via ingestion or circulation, act by passive diffusion or active targeting using markers and local stimuli [59,60]. Alternatively, depot systems such as films and gels enable localized, sustained release near tumors [60].

### 3.2. Routes of Administration

**Figure 4:**
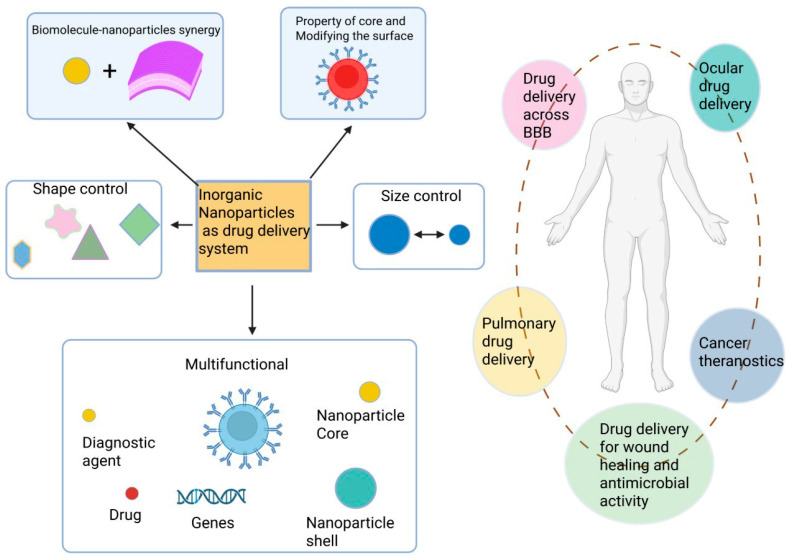
Routes of administration of inorganic nanoparticles into the body. Reproduced, with permission, License CC BY 4.0, from [75].

The administration route of inorganic nanoparticles significantly affects their therapeutic efficacy, biodistribution, and safety profile [88]. Major routes include oral, parenteral, interstitial, nasal, topical, transdermal, inhalation, and others (Figure 4). Each has distinct benefits and challenges that must be considered in drug design.

Oral administration enhances solubility and stability in the gastrointestinal tract (GIT) and is effective for treating GIT diseases such as inflammatory bowel disease and colorectal cancer [89]. However, challenges include enzymatic degradation, pH variation, and mucosal barriers.

Parenteral administration (intravenous or subcutaneous) bypasses the GIT and ensures rapid systemic distribution [90]. It allows targeted delivery with reduced side effects but requires careful surface engineering.

Interstitial administration enables delivery from peripheral tissues to lymph nodes through intramuscular or subcutaneous injections, facilitating lymphatic targeting [91].

Inhalation delivers nanoparticles directly to the lungs, offering rapid absorption and treatment for respiratory diseases, though particle size and deposition efficiency are critical [92,93].

Nasal administration provides rapid absorption and direct access to the CNS, bypassing the blood–brain barrier [94].

Topical and transdermal routes target skin or localized tumors, enhancing penetration through the skin barrier and enabling sustained release via nanoparticle-loaded patches [83,95,96,97,98].

Once administered, nanoparticles must overcome biological barriers including opsonization, endothelial transport, extracellular matrix penetration, and cellular uptake [99,100,101]. Clearance by the reticuloendothelial system (RES), especially liver and spleen, limits systemic availability. Particle size and surface properties strongly influence biodistribution and elimination. Nanoparticles smaller than ~5.5 nm are primarily cleared renally [102,103,104], while larger or non-biodegradable particles are retained in the liver or cleared hepatobiliary, Table 4. The optimal size range for tumor targeting is ~40–60 nm, balancing clearance avoidance and penetration efficiency [105,106].

Inorganic QDs also display route-dependent biodistribution: oral delivery often reduces bioavailability due to degradation, IV injection leads to rapid systemic distribution with liver/kidney accumulation [106], interstitial delivery enhances localized targeting, inhalation penetrates pulmonary tissue, and topical application supports localized therapy with limited systemic uptake.

Surface modifications, such as PEGylation, can reduce opsonization and prolong circulation [66,67,107]. Without modification, QDs rapidly attract plasma proteins, forming a “protein corona” that accelerates clearance by macrophages in the liver and spleen, reducing therapeutic availability. Proper engineering of surface chemistry and route of administration is thus essential for improving QD circulation, biodistribution, and cancer-targeting efficacy.

### 3.3. Targeting Strategies

**Figure 5:**
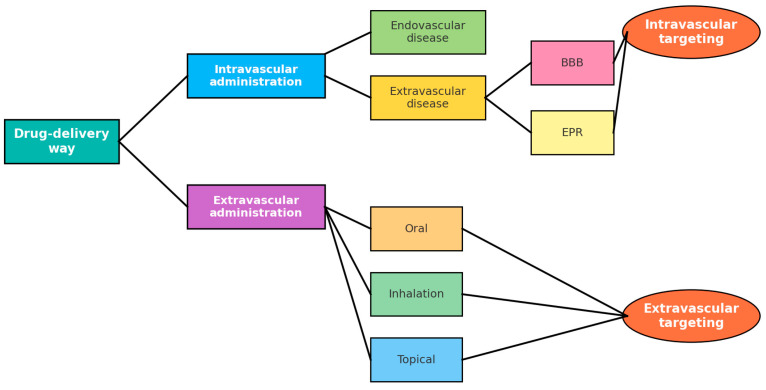
Flowchart of different routes of administration and endovascular targeting Vs. extra vascular targeting. Reproduced and redesigned, open access, license CC BY 4.0, from [109].

Nanoparticle targeting represents an innovative approach in drug delivery systems, especially in the treatment of cancer [108]. The aforementioned method exploits the unique properties of nanoparticles to improve the efficacy and specificity of the therapeutic agents while minimizing side effects. Quantum dots typically range in size from 1 to 10 nanometers, allowing them to effectively cross biological barriers. Their size can affect their circulation time and ability to penetrate tissues. Nanoparticle surfaces can be modified with antibodies, ligands, or peptides that explicitly attach to receptors exaggerated on cancer cells, enhancing active targeting capabilities. Several targeting mechanisms have been explored. Passive targeting: This approach exploits the increased permeability and retention (EPR) effect, where nanoparticles accumulate in tumor tissues due to their leaky vasculature and inadequate lymphatic drainage [68]. This method does not require specific target agents. Active targeting: comprises attaching specific ligands to the nano surface that attach to receptors on target cells, facilitating selective uptake by cancer cells. This method improves the precision of drug delivery compared to passive targeting. Magnetic targeting utilizes the unique properties of magnetic nanoparticles (MNPs) to be directed to particular areas of the body using external magnetic fields. This allows for increased accumulation of nanoparticles at tumor sites, improving localized treatment and reducing systemic drug exposure (Figure 5).

## 4. Improve Targeting Ability and Therapeutic Properties of QD Heterostructure

Biomolecule-derived quantum dots exhibit enhanced stability and biocompatibility versus conventional metal-based quantum dots. Their advantageous characteristics render them particularly compatible for various biomedical uses, such as drug delivery and bio-imaging, while mitigating the toxicity issues linked to metal-based options. As research advances, the growth of these environmentally friendly nanomaterials is anticipated to broaden into multiple domains that require safe and efficient fluorescent probes. Consequently, various molecules, including folic acid (FA), proteins, nucleic acids, and amino acids, are utilized to enhance the targeting capabilities of quantum dots [71,72,73], Table 5.

### 4.1. Folate Receptors (FR)

Folic acid (FA) plays an important role in regulating the metabolic processes that are vital for the human body. FA receptor (FR) over-expression has been mentioned for many cancers, but there is still limited or conflicting data regarding folate receptors (FRs) in breast cancer cells. Spacers are flexible molecules used in bioconjugate chemistry due to their properties of non-toxicity, biodegradability, and compatibility. They correlate with linkers and other bioconjugates like folic acid and therapeutic agents. The effective release of stimuli-responsive NCs and SMDCs depends upon internal and external stimuli, such as pH, enzymatic acidity, glutathione, hypoxia, and redox potential changes, which promote the regulated degradation of drug transporters into their constituent parts for effective drug delivery (B). Alibolandi et al. employed a tumor-targeted quantum dot (QD) system to actively deliver doxorubicin (DOX) coated in a polyethylene glycol-poly(lactic-co-glycolic acid) (PEG-PLGA) formulation [74,75,118,119]. The QD and DOX-loaded nanoparticles were integrated with folate to facilitate receptor-guided delivery, targeting folate-binding protein receptors that are over-expressed in different cancer cells, thereby enhancing the specificity of cancer targeting. The nanopolymersomes’ bilayer and core were designed to contain hydrophilic MSA-capped QDs and hydrophobic DOX, respectively. In vivo studies demonstrated that six hours post-intravenous injection, the folate receptor-targeted QD-encapsulated nanoparticles accumulated at tumor sites in BALB/c mice with 4T1 breast adenocarcinoma, as confirmed by the method of whole organ tissue homogenate analysis and organ fluorescence microscopy imaging. Furthermore, evaluations of acute toxicity indicated that the targeted quantum dot-based nanoparticles never produce any long-term adverse histopathological or physiological effects on the treated animals [109,120,121,122].

A different investigation by Yang et al. represented the application of hydrophilic CdTe quantum dots (QDs) encapsulated within folate receptor (FR)-targeted liposomes as luminescent probes for live cells imaging. The process involved hydrating a lipid thin film with a CdTe solution to form FR-targeted QD liposomes, with the hydrophilic CdTe QDs being produced directly in the aqueous phase [123,124]. The formulations were characterized using zeta potential measurements, liposomal particle size analysis, and UV-visible and fluorescence spectroscopy. HeLa cells, a cell line of human cervical cancer, were employed to evaluate the targeting and imaging capabilities of the FR-targeted liposomes. Additionally, the cytotoxicity of these quantum dot liposomes was assessed by treating HeLa cells with FR-targeted, non-targeted, and free QD liposomes [125,126,127]. Monteiro, C.A., and colleagues examined the assimilation and recycling of FRs in breast cancer cells by employing QDs merged with folic acid (FA), using HeLa cells as a control group. The quantum dots were connected covalently to folic acid under varying conditions, and the most effective conjugate was selected for studying folic receptors in MCF7, T47D, MDA-MB231, and HeLa cell lines through confocal microscopy and flow cytometry [128,129]. The specificity and efficiency of the conjugation were assessed through fluorescence correlation spectroscopy (FCS) and saturation assays, with FCS confirming successful conjugation [130]. Results showed that HeLa and T47D cells internalized a significantly elevated percentage of FRs (95% and 90%, respectively) compared to MDA-MB231 cells (68%), while MCF7 cells had minimal functional FR levels (3%). It is indicated by the Saturation assays that the QD-FA conjugates were specific and also revealed generally low recycling rates of FR across most cell types studied, except for T47D. Overall, the effective development of QD-FA conjugates suggests that therapies that targeting the FRs may be particularly beneficial for T47D, MDA-MB231, and HeLa cells [116,131,132].

### 4.2. Transferrin Receptor (Tfr)

Tf is a protein recognized for its high binding affinity to the transferrin receptor (TfR), that is often overproduced in cancer cells. In a study conducted by Yong, K.T. et al., techniques like confocal and two-photon fluorescence imaging were employed to verify the receptor-specific absorption of QR-Tf conjugates in HeLa cells, recognized for their high TfR expression [132,133]. To target optical probes, the research utilized the synthesized CdSe/CdS/ZnS quantum rods in live cell imaging process. These synthesized quantum rods were produced via applying a graded shell of CdS/ZnS onto the core of CdSe rods within a surfactant solution [134]. To improve their solubility in dimethyl sulfoxide (DMSO), the partially polar surfactant mercaptoundecanoic acid (MUA) replaced the hydrophobic surfactants on the nanorods’ surface. Subsequently, lysine was utilized to cross-link the groups of carboxylic acid present in MUA on the QR surface via carbodiimide chemistry, resulting in a hydrophilic shell containing both carboxyl and amine groups. Transferrin (Tf) was subsequently conjugated with this surface employing the same carbodiimide chemistry, forming Quantum Rods-Transferrin bioconjugates [135,136,137].

### 4.3. Aptamers (DNA, siRNA)

Aptamer-targeted quantum dots (QDs) signify a major breakthrough in biosensing, imaging, and targeted cancer therapies. These nanomaterials merge the distinctive optical characteristics of QDs having the specificity of short as well as single-stranded nucleic acids, aptamers, which adhere to specific targets with high affinity [138,139]. The metastatic recurrence of hepatocellular carcinoma (HCC) is a critical biological behavior also the primary reason of treatment failure. Currently, early diagnosis of metastasis is not feasible because of the absence of specific molecular probes capable of identifying metastatic HCC cells. Recently, aptamers have evolved as potential candidates for molecular probes in biomedical applications [140,141].

**Figure 6:**
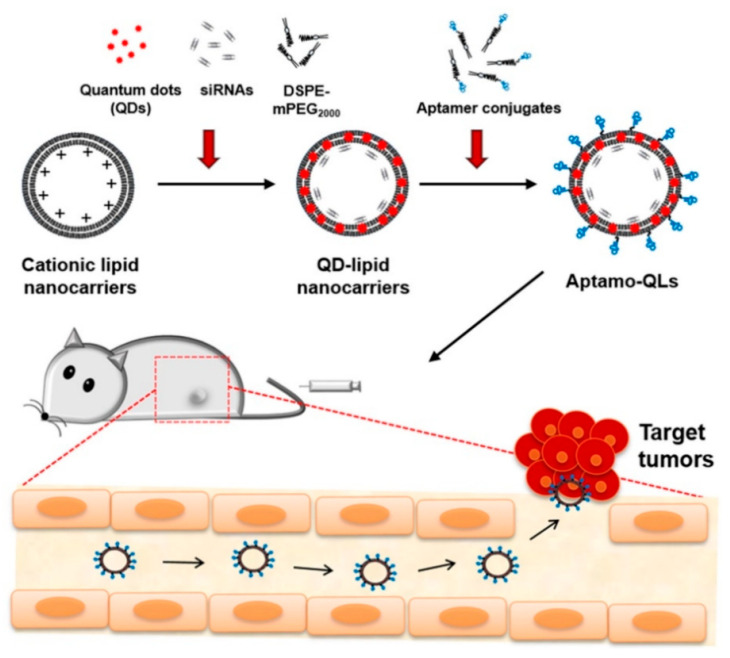
Illustration of the theranostic strategy for RNAi gene therapy and fluorescence tumor imaging. Anti-EGFR aptamer-conjugated lipid nanocarriers encapsulating QDs and anti-cancer siRNAs were fabricated and intravenously administered. The administered lipid nanocarriers presumably extravasated through the leaky tumor vasculature and then targeted primary tumors via specific recognition of EGF receptors overexpressed on MDA-MB-231 tumors. The delivered QDs and therapeutic siRNAsprovide fluorescence tumor images and inhibitory effects on tumor growth. Reproduced with open access, license: CC BY-NC 4.0 from [152].

Drawn from the properties of multivalent binding of streptavidin (SA) to biotin, Zhang, M.Z. et al and researchers designed a quantum dot probe with multifunctional properties, referred to as QD-[AS-ODN+p160), which incorporates an antisense peptide p160 and oligonucleotide (AS-ODN). This probe is intended for real-time monitoring of the targeted delivery of AS-ODN and to regulate the folate receptor-α (hFR-α) in breast cancer cells, MCF-7 [142]. Confocal imaging and flow cytometry demonstrated that QD-[AS-ODN+p160] specifically targets MCF-7 breast cancer cells. Low-temperature and ATP-depletion experiments indicate energy-dependent uptake, and co-localization studies confirm receptor-mediated endocytosis as the primary internalization pathway. These results show that QD-[AS-ODN+p160] enables cell-selective gene silencing and real-time tracking of AS-ODN delivery [143]. Wang et al. conducted research on the aptamers identification that specifically target metastatic hepatocellular carcinoma (HCC) cells. They utilized two HCC cell lines: HCCLM9, known for its prominent metastatic capability, and MHCC97-L, which has limited metastatic potential [144]. Using whole-cell SELEX with HCCLM9 as the target and MHCC97-L as the control, six candidate aptamers were generated, among which LY-1 showed high affinity and specificity for metastatic HCC cells [145]. This was confirmed through experiments involving cell cultures, animal models of HCC metastasis, and clinical samples. Notably, when conjugated with quantum magnetic particles, LY-1 demonstrated a capacity to effectively seize HCC cells from the mixture of complexes just like whole blood. These outcomes mention that LY-1 could function as a valuable molecular probe for the detection of metastatic HCC cells [146]. In another approach, Saharkhiz, S. et al and researchers utilized modified MSNs, specifically by coating their surfaces with a thermo-responsive cationic lipid functionalized with an anti-PSMA aptamer, to facilitate selective administration of paclitaxel (PTX) and CdSe/ZnS quantum dots (QDs) to prostate cancer cells that express PSMA. The MSNs, QDs, and lipid coatings were synthesized using thin film hydration, hot injection, and sol-gel techniques respectively [147]. The study confirmed the successful fabrication of Apt-L-MSNs (~150 nm, spherical) encapsulating PTX and QDs (~6 nm) with 88% efficiency. The release profile showed sustained PTX release, nearly doubling at 42 °C compared to 37 °C. MTT assay and fluorescence microscopy revealed enhanced uptake by LNCaP cells, achieving ~80% cell degradation, superior to pure PTX. These results highlight the potential of this system for biomedical applications [148,149]. Numerous aptamers can be used for their potential as drug delivery systems to target specific ligands, as well as various interfering RNAs (siRNAs) of small size have been investigated for their carcinostatic effects. Though, the similar physicochemical characteristics of these two types of molecules have made it challenging to design aptamer-guided carriers for siRNA encapsulation [150]. In this study, Kim et al. developed aptamer-linked lipid nanocarriers encapsulating quantum dots (QDs) and siRNAs for theranostic of triple-negative breast cancer (TNBC). Hydrophobic QDs were integrated into lipid bilayers, while therapeutic siRNAs were coordinated with QD–lipid nanocarriers (QLs). Anti-EGFR aptamer–lipid conjugates were incorporated to produce TNBC-targeted aptamo-QLs. The study directly compared these with anti-EGFR antibody–coupled immuno-QLs, and in vitro delivery of siRNAs and QDs was assessed using confocal microscopy and flow cytometry [151] (Figure 6).

Ag_2_S quantum dots (QDs) exhibit excellent NIR-II optical properties and favorable biocompatibility, but their limited targeting and low solubility require modification to enhance theranostic applications [151]. Huang et al. employed rolling circle amplification (RCA) to generate linear ssDNA containing a PD-L1 aptamer and a C-rich palindromic sequence, enabling selective Ag2+ chelation and biomimetic formation of pApt-Ag2S QDs [153]. These QDs selectively target tumors with high PD-L1 expression, provide NIR-II photothermal capabilities, and inhibit PD-L1-mediated immunosuppression, creating an integrated photothermal therapy and immune checkpoint blockade platform. Long ssDNA templates further improved their photostability and biological resistance, yielding effective in vitro and in vivo theranostics [154,155,156].

Another study developed PEG-PCL nanopolymersomes encapsulating Gd-based QDs and Doxorubicin (DOX) for MR-fluorescence imaging and anticancer therapy. Hydrophobic QDs and DOX were co-loaded using a double emulsion technique [157]. Surface conjugation with DNA aptamer AS1411 enhanced cellular uptake and cytotoxicity in nucleolin-overexpressing cells (*p* < 0.05), and improved tumor inhibition and survival in 4T1 tumor-bearing mice (*p* < 0.05) [110,111].

siRNA delivery remains challenging due to difficulty in tracking transfection efficiency. Tan et al. developed chitosan nanoparticles with surface-encapsulated QDs for HER2/neu siRNA delivery. HER2-targeted QD-chitosan NPs enabled selective siRNA delivery to SKBR3 cells, with gene-silencing confirmed by HER2 ELISA and luciferase assays. This self-tracking design facilitates monitoring of in vivo gene silencing [112].

### 4.4. αvβ3 Integrin

Mulder et al. present research on a distinct type of QD-based nanoparticle specifically engineered to target the αvβ3 integrin, enhancing its capabilities for optical as well as magnetic resonance imaging of tumor angiogenesis. After intravenously administering RGD-conjugated quantum dots (RGD-pQDs) to tumor-bearing mice, intravital microscopy employed for observing activated endothelial cells involved in angiogenesis at a cellular level, despite challenges related to limited scanning area and depth of penetration. Concurrently, magnetic resonance imaging facilitated anatomical visualization of angiogenesis throughout the tumor mass, while fluorescence imaging allowed for a thorough evaluation of angiogenic activity across the entire body. By integrating these QDs with the previously mentioned imaging techniques, researchers successfully identified the tumor vasculature associated with angiogenesis, observing the highest levels of activity at the tumor’s periphery. This innovative nanoparticle shows promise for multimodal imaging applications in various diseases characterized by endothelial cell activation [113,114].

### 4.5. Hyaluronic Acid (HA)

**Figure 7:**
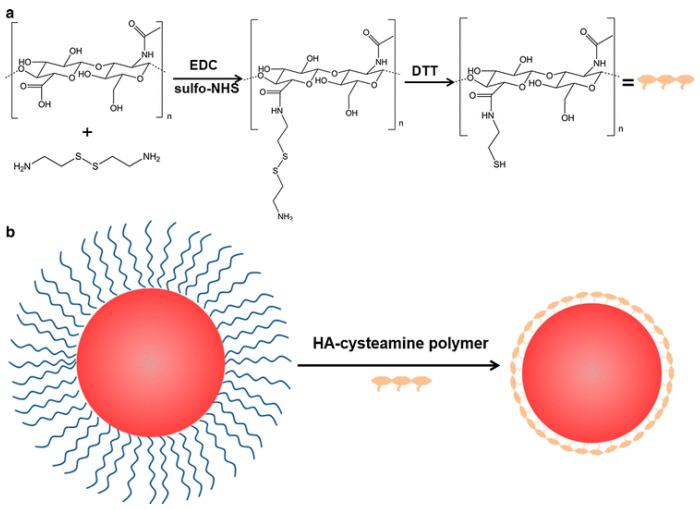
Fabrication of HA-cysteamine polymer-coated QDs. (**a**) Synthesis of HA-cysteamine polymer. (**b**) HA polymer coating on QDs. Reproduced with permission from [158].

Wang et al. developed cysteamine-modified HA to encapsulate QDs via a one-step reverse micelle method, yielding QDs of ~22.6 nm [114]. The HA coating provided excellent stability in PBS for over 140 days, tolerated pH 2–12, and maintained strong fluorescence in BSA-containing cell culture media. Cell assays showed minimal cytotoxicity in MD-MB-231 breast cancer cells and confirmed CD44-mediated targeting, validated by an HA competition experiment (Figure 7).

Polymer-drug conjugates improve tumor targeting and the specificity of anticancer agents. In one study, quantum dots and melphalan were linked to a hyaluronic acid framework to form a polymer-drug conjugate [112]. Characterization using UV-Vis, FT-IR, XRD, ^1^H NMR, and DLS confirmed self-assembly into nanoparticles of 115 ± 2.3 nm. The conjugate exhibited pH-sensitive controlled drug release and receptor-mediated uptake by human breast cancer cells, showing stronger cytotoxicity toward cancer cells than normal cells. These results suggest its potential as an effective cancer therapeutic platform.

Accurate identification of cancer cells using fluorescence is crucial for cancer diagnosis. In this study, we developed blue, fluorescent nitrogen-doped graphene quantum dots (N-GQDs) from diethylamine and citric acid and through a straightforward single-step hydrothermal synthesis method, that minimizes by-products and emphasizes binding sites for precise targeting. The incorporation of nitrogen resulted in a significant presence of amide II bonds, creating numerous binding sites for conjugation with hyaluronic acid (HA). We then conjugated N-GQDs at varying pH levels to HA via amide bonds, with basic conditions proving more promising for bond formation. The HA-conjugated N-GQDs (HA-N-GQDs) are designed to bind alongside CD44, that is over-expressed on the surface of MCF-7 breast cancer cells, leading to enhanced fluorescence in these cells. HA-N-GQDs demonstrated strong fluorescence intensity, less toxicity, together with excellent cytocompatibility, positioning them as effective agents for fluorescence imaging and accurate cancer cell identification [113].

Hyaluronic acid (HA), a natural polysaccharide, is widely used in drug delivery but suffers from low drug-loading capacity and leakage during circulation. To improve efficiency, porous silica (pSiO_2_) nanocarriers modified with HA have been developed, combining the high loading capacity of pSiO_2_ with HA’s targeting ability. In one study, Ag_2_S quantum dots (QDs) were embedded within pSiO_2_ carriers (~30 nm) to provide photothermal properties, achieving a drug-loading capacity of 29.3% [114]. HA was attached to the carrier surface via a disulfide-linked alkyl amine connector and amide bond formation after DOX loading, effectively sealing the drug. The HA–CD44 interaction enhances cancer cell targeting, and hyaluronidase-mediated HA degradation triggers controlled drug release, enabling responsive photothermal chemotherapy.

A novel fluorescent sensing system has been created that is both hypersensitive and capable of bidirectional detection. This arrangement identifies cisplatin prodrug cross-linked hyaluronic acid (CPHA) hydrogels, which act as quenchers, and DNA, serving as receptors, utilizing mercaptopropionic acid (MPA) capped cadmium telluride (CdTe) quantum dots (QDs).

In contrast to earlier research on this platinum-based chemical, this study uses CPHA hydrogel as a multifunctional delivery strategy to address medication resistance and serious side effects. The manufacture of the cisplatin prodrug and the photo-induced electron transfer-induced fluorescence quenching of CdTe QDs serve as foundation for the recognition process.

In the CdTe QD–CPHA hydrogel system, DNA linked with platinum ions restores fluorescence in a reversible “turn off–on” manner. This enables sensitive DNA quantification, with a detection range of 10–50 nM and 1.50 nM LOD for CPHA hydrogel, and 0–75 nM with 0.60 nM LOD for DNA. This approach supports platinum-based drug delivery with reduced side effects, efficient anticancer drug screening, and precise low-concentration DNA detection [117].

**Figure 8:**
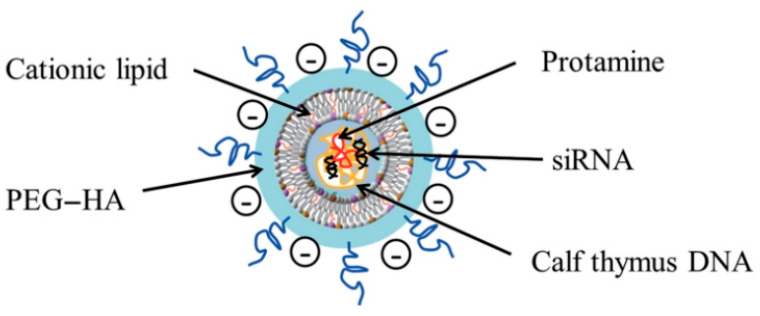
Representation of PEG–HA–NP. Briefly, naked nanoparticles (NPs) were generated via electrostatic interaction between cationic liposomes and siRNA, with protamine and calf thymus DNA facilitating siRNA condensation. Subsequently, the naked NP was rapidly mixed with a PEG–HA solution at a 1:2 volume ratio under vigorous vortexing to form PEG–HA–NP. Reprinted with permission from Elsevier [161].

Chemotherapy is often limited by poor targeting and side effects. Multifunctional delivery systems have emerged to improve efficacy. A study developed niosomes (NIO) co-loaded with paclitaxel (PTX) and sodium oxamate (SO), incorporating QDs for bioimaging and hyaluronic acid (HA) for targeting as reported in Figure 8. The resulting HN@QPS nanoparticles (~150 nm, −39.9 mV, >90% PTX encapsulation) enhanced anticancer activity, achieving IC50 values of 1–5 ppm for HN@QP and >0.5 ppm for HN@QPS. Treatment increased apoptosis in MCF-7 cells by >70% while showing minimal toxicity to normal HHF-2 cells. Cellular uptake studies confirmed improved internalization, and mitochondrial fluorescence indicated effective cytotoxicity [159]. In another study, anti-GGCT siRNA was delivered via PEG–HA-modified liposomal nanoparticles (PEG–HA–NP) to target drug-resistant MCF-7 cells. The nanoparticles (~216 nm, −17.4 mV) efficiently downregulated GGCT, inducing cytotoxic effects in MCF-7/ADR cells. Systemic administration at 0.35 mg/kg siRNA inhibited tumor growth and induced necrosis without significant toxicity to normal tissues, demonstrating the potential of PEG–HA–NP for managing drug-resistant breast cancer [160].

### 4.6. Antibody (Ab)

Although immunohistochemical techniques and additional methods for diagnosis and early detection of breast cancer and lung cancer biomarkers are widely accessible, diagnosing these cancers in their early stages can be challenging and often leads to inaccuracies. There is a critical requirement to detect and validate early biomarkers that are particular to lung and breast cancers, as this might enable the creation of more precise detection methods for the onset of these diseases. Tatsiana Y. Rakovich, et al., investigated, ultra-small along with luminescent nanoprobes composed of quantum dots (QDs) linked to single domain anti-HER2 antibodies (sdAbs) that are utilized for the immunolabeling of the cell lines of lung and breast cancer. Their efficacy has compared with same type of conventional anti-HER2 monoclonal antibodies bonded to organic dyes, specifically Alexa Fluor 488 and Alexa Fluor 568. The sdAbs-QD conjugates demonstrated enhanced staining capabilities across various lung cancer cell lines exhibiting different levels of HER2 expression that indicates their significant capability for creating more sensitive assays aimed at the early detection of cancer biomarkers [162].

The involvement of tumor stroma to regulate the growth of breast cancer has been extensively researched. Nonetheless, the specifics regarding the nature of heterocellular interactions between breast cancer cells (BCCs) and stromal cells remain inadequately understood. In his Pietilae, M., et al. aimed to explore the intercellular communication between human mesenchymal stromal cells (hMSCs) and breast cancer cells (BCCs, specifically MDA-MB-231). For this purpose, utilized cell-internalizing quantum dots (i-QD), which were created by linking a cell-internalizing anti-mortalin antibody with QDs. Co-culturing color-coded and Luminous hMSCs (QD655) with BCCs (QD585) demonstrated the transfer of the QD655 signal from hMSCs to BCCs. Over a period of 48 h in co-culture, the number of QD double-positive BCCs exhibited a steady rise over time.

Notably, significant intercellular transfer of QD655 was observed in a hanging drop co-culture system, while it observed absent in a trans-well system that did not allow direct cell-to-cell contact. Analyses using fluorescent and electron microscopy further indicated that direct interactions between cells are likely necessary for the QD655 transfer from hMSCs to BCCs [163].

Semiconductor quantum dots are an innovative class of fluorophores with distinct physical and chemical properties, enabling the outstanding increase of the latest applications of optical diagnostics and fluorescent imaging (Table 1). In another approach, Zdobnova, T.A., and colleagues found the capacity of quantum dot-antibody complexes as effective agents for the fluorescent visualization of specific biomarkers that are overexpressed in tumor tissues. They later detailed the development of self-configuring fluorescent complexes using QDs in conjunction with antibodies like anti-HER1 or anti-HER2/neu scFv, examining their interrelation with cultured tumor cells. A binding approach leveraging a well-defined non-covalent interaction of the proteins barnase and barstar employed to link the QDs with the targeting antibodies. This method enables the integration of targeting and visualization capabilities by simply adjusting the relevant components of the fluorescent complex [164].

Wu et al. developed a dual-signal amplification immunosensor for sensitive and specific detection of rare cancer cells [165]. Graphene-modified electrodes enhanced electron transfer, while QD-coated silica nanoparticles served as tracers for two biomarkers. Capture antibodies immobilized on chitosan–reduced graphene oxide films enabled a sandwich immunoreaction, producing distinct voltametric peaks corresponding to antigen identity and quantity. The method successfully detected GPC3 and EpCAM on Hep3B liver cancer cells, demonstrating high sensitivity, specificity, stability, and reproducibility, highlighting its potential for molecular and clinical diagnostics.

Kim et al. developed an antibody-conjugated QD nanoprobe to assess targeting specificity in a melanoma–melanocyte coculture using automated confocal microscopy. Antibody conjugation enhanced melanoma-specific binding, while unconjugated QDs showed non-specific melanocyte interaction. Concentration-dependent and competitive inhibition assays confirmed melanoma selectivity. This coculture model provides a rapid, sensitive platform for in vitro melanoma detection and may aid high-throughput cancer screening and therapy development [166].

### 4.7. Anti EGFR

A quantum-dot (QD)-incorporated micelle functionalized with an anti-epidermal growth factor receptor (EGFR) nanobody (Nb) and loaded with an anticancer drug, aminoflavone (AF), has been designed for targeted theranostic of EGFR-overexpressing cancers. The indium phosphate core/zinc sulfide shell quantum dots (InP/ZnS QDs) exhibited near-infrared (NIR) fluorescence, enabling in vivo studies on nanoparticle biodistribution. The anti-EGFR nanobody 7D12 conjugation refined the cellular absorption and cytotoxicity of the micelles based on quantum dots in EGFR-overexpressing MDA-MB-468 triple-negative breast cancer (TNBC) cells. In contrast to the AF-coated nontargeted (i.e., absent of Nb conjugation) micelles, the AF-coated Nb-conjugated (i.e., targeted) micelles aggregated in tumors in increased amount, resulting in more efficient tumor regression in an orthotopic triple-negative breast cancer xenograft mouse model. Moreover, there was no systemic toxicity detected with the treatments. Therefore, this QD-based Nb-conjugated micelle may function as a prominent theranostic nanoplatform for EGFR-overexpressing cancers such as TNBCs [167].

### 4.8. Peptide

**Figure 9:**
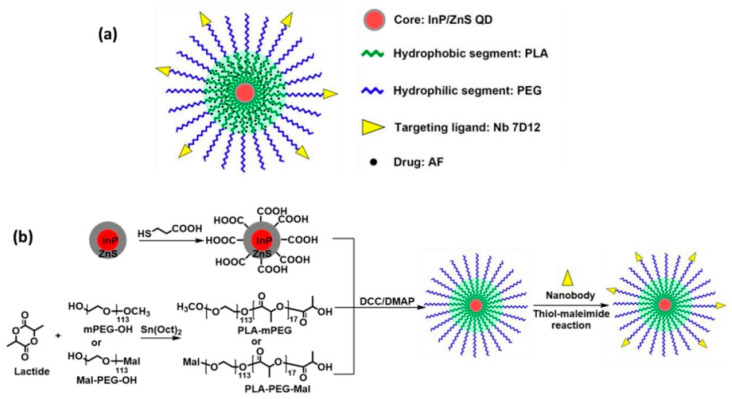
Representation of (**a**) QD-PLA-PEG Micelles Conjugated with 7D12 Nb and (**b**) Synthesis Chart for QD-PLA-PEG-Nb [169]. Reprinted with permission from: Copyright 2017 [169].

Anobiomaterials can be designed to detect and target cancer-specific receptors at the cellular level, for both diagnostic and therapeutic applications. This study presents the fabrication of newly researched multifunctional nanocomposites formed by fluorescent inorganic semiconductor quantum dot (QD) cores and tripeptide-modified polysaccharide organic shells. These structures were engineered for targeting as well as imaging the cancer cells α_v_β_3_ integrin receptors. Primarily, chitosan was covalently bound with the RGD peptide by utilizing a crosslinker to form bioconjugates (RGD-chitosan), which were then applied as capping ligands to facilitate the single-step synthesis of surface-functionalized CdS QDs in aqueous media at room temperature. These core-shell nanostructures were thoroughly characterized by Fourier transform infrared spectroscopy (FTIR), UV–vis spectroscopy, dynamic light scattering (DLS), photoluminescence [PL) spectroscopy, transmission electron microscopy (TEM), and zeta potential (ZP). The images from TEM and the graphs of UV–vis absorption spectra indicated the synthesis of ultra-small CdS quantum dots nanocrystals with average scale of size 2.0 to 3.0 nm. Furthermore, the PL results indicated that the nanobioconjugates represented strong green fluorescence under excitation. The CdS-RGD-chitosan systems were efficient at selective targeting integrin when tested precisely in vitro applying two model cell cultures, a non-cancerous human embryonic kidney cell, HEK 293 and cancerous sarcoma osteogenic-derived cells, SAOS imaging by fluorescence microscopy (Figure 9) [167,168].

## 5. Targeted Combination Therapies Using Different QDs

Targeted combination therapy utilizing nanomaterials indicates a remarkable development in the treatment of cancer, increasing therapeutic potency when reducing side effects. This approach integrates nanoparticles (NPs) with chemotherapeutic agents to achieve synergistic effects, improve drug delivery, and address challenges such as drug resistance [152]. Nanoparticles can encapsulate multiple drugs, ensuring for regulated release and targeted delivery to tumor sites. This is achieved through mechanisms like the improved permeability and retention effect (EPR), that supports nanoparticles to deposit in tumors as a result of their leaky vasculature [164]. Additionally, active targeting strategies can be employed by conjugating NPs with ligands which specifically bind to over-expressed receptors on cancer cells [170]. Combination therapy leverages the synergistic actions of different drugs, which may target various routes associated in the growth of tumor and their survival. This multifaceted strategy not only increases therapeutic outcomes but also helps mitigate the development of drug resistance that often occurs with mono therapy [170]. For instance, the co-delivery of chemotherapeutics using multifunctional NPs has been shown to improve treatment efficacy compared to single-agent therapies [171]. By using lower doses of multiple drugs delivered precisely to the tumor site, combination therapy via NPs can reduce systemic toxicity and adverse effects associated with higher doses of single agents [172,173]. This targeted approach allows for a more favorable therapeutic index.

### 5.1. Chemotherapy (CHT)-Photodynamic Therapy (PDT)

Sensitization is a key factor in photodynamic therapy (PDT) for cancer and other diseases, and the integration of quantum dots (QDs) with PDT photosensitizers offers new avenues for enhancing therapeutic efficacy. QDs can facilitate the generation of singlet oxygen through QD-organic interactions, yet their full potential in biological systems remains underexplored [174]. Ahirwar et al. demonstrated the use of graphene quantum dots (GQDs) and graphene oxide quantum dots (GOQDs) in PDT to efficiently eliminate cancer cells. GQDs synthesized via electrochemical exfoliation of graphite rods (1.5–5.5 nm) showed strong UV absorbance, excitation-dependent photoluminescence across UV and visible spectra, and singlet oxygen generation. Impressively, over 90% of cancer cells were destroyed after only five minutes of exposure, while over 80% of untreated cells survived the same irradiation period. Advantages of GQDs/GOQDs include high cytotoxic efficiency under low-power UV light, short irradiation times, and uniform treatment across large areas. However, their use is largely limited to skin cancers due to shallow UV light penetration [174].

Traditional photosensitizers in PDT face challenges such as poor solubility, photoinstability, and aggregation. To address these, Murali et al. developed hematoporphyrin (HP)-encapsulated carbon quantum dots (CQDs) via a single-step microwave-assisted method using HP monomer as a precursor. The resulting HP-CQDs preserved HP’s chemical and optical properties while exhibiting improved water solubility. They generated reactive oxygen species under deep red light, achieving enhanced PDT efficacy against MCF-7 human breast cancer cells. Compared to free HP, HP-CQDs displayed higher phototoxicity and lower dark toxicity [175,176,177].

While GQDs and GOQDs enable rapid and highly efficient photodynamic therapy (PDT) under UV light, their clinical use is largely limited to superficial tumors due to the shallow penetration of UV radiation. In contrast, carbon quantum dot (CQD)-based photosensitizers, such as hematoporphyrin-encapsulated CQDs (HP-CQDs), can be activated by deeper-penetrating red light, allowing broader applicability for treating internal cancers while reducing off-target side effects. Despite these advantages, both approaches face challenges, including concerns over long-term biocompatibility, potential in vivo toxicity, and difficulties in large-scale synthesis. Future research should prioritize optimizing quantum dot size, surface functionalization, and hybridization with photosensitizers to enhance tissue penetration, increase reactive oxygen species (ROS) generation efficiency, and achieve controlled biodistribution, thereby advancing the clinical translation of PDT.

Semiconductor nanoparticles, commonly referred to as quantum dots (QDs), have been recognized as promising candidates for photodynamic therapy (PDT) since 2003. Research work involving cadmium-based QDs has demonstrated promising outcomes when used alongside molecular photosensitizers. Though, issues related to the toxicity of these quantum dots and the overall less effectiveness of these combinations remain a concern. In a study by Charron et al., two different types of less-toxic quantum dots of InP/ZnS were combined with the photosensitizer chlorin e6, leading to an in-depth analysis of the spectroscopic characteristics of these hybrids. Various spectroscopic methods were utilized to elucidate the mechanisms of energy transfer, kinetics, and the rate at which singlet oxygen is generated among all components. Moreover, the PDT potential of the QD/chlorin e6 hybrids was evaluated against the MDA-MB-231 breast cancer cell line using a colorimetric MTT assay. The results indicated that energy transfer between QDs and the molecular photosensitizer is a crucial limiting factor for singlet oxygen production, demonstrating that cell viability rates for both the hybrid and free photosensitizer were comparable. These comprehensive insights underscore that the transfer of energy in-between QDs and photosensitizers serves as a “bottleneck,” indicating that enhancing the chemical design of QD/photosensitizer hybrids in future research is imperative [178].

### 5.2. CHT-Radiotherapy (RDT)

A different strategy involves the use of multimodal nanoparticles composed of gold nanoparticles (Au NPs), mesoporous silica nanoparticles (MSNs), and quantum dots (QDs), as demonstrated by Abrishami et al. This method shows considerable promise for advanced drug delivery systems tailored for targeted cancer therapy and imaging. The process includes encapsulating magnetic GZCIS/ZnS QDs within mesoporous silica, incorporating the chemotherapeutic agent epirubicin into the silica’s pores, layering with Au NPs, PEGylation, and conjugating with epithelial cell adhesion molecule (EpCAM) aptamers to specifically target colorectal cancer (CRC) cells. The study details the characteristics of the hybrid QD@MSN-EPI-Au-PEG-Apt nanocarriers, which are approximately 65 nm in size after synthesis. In vitro tests indicate that these targeted nanocarriers demonstrate selective cytotoxicity aimed at HT-29 cells in relation to CHO cells, contributing to a notable reduction in HT-29 cell viability when used in conjunction with radiation therapy. The effectiveness of these nanocarriers for targeted delivery in vivo is corroborated through improved anti-tumor responses and minimized side effects following chemo-radiotherapy, along with imaging capabilities observed in a CRC mouse model. This innovative approach offers possibilities for enhancing theranostic outcomes in the treatment of colorectal cancer [158].

### 5.3. Photothermal Therapy-(PTT)-PDT

Combining photodynamic therapy (PDT) and photothermal therapy (PTT) provides a promising strategy for cancer treatment by leveraging their complementary mechanisms to enhance efficacy and reduce side effects. PDT utilizes photosensitizers activated by specific light wavelengths to generate reactive oxygen species (ROS), inducing direct tumor cell death and damaging tumor vasculature, which is particularly effective for surface-level tumors due to limited light penetration and ROS diffusion. In contrast, PTT employs near-infrared (NIR) light-absorbing agents to generate localized hyperthermia, directly killing cancer cells and improving blood flow, which can enhance oxygen delivery and sensitize tumors to PDT [158,179,180].

The combination of PDT and PTT offers several advantages: PDT can modulate the tumor microenvironment (TME) to increase thermal sensitivity, while PTT-induced hyperthermia improves oxygenation, crucial for ROS-mediated PDT. Both therapies can also trigger immunogenic cell death (ICD), releasing tumor-associated antigens to stimulate systemic immune responses [168]. However, limitations exist, including restricted light penetration for deep-seated tumors, potential off-target heating, and the challenge of optimizing dose and timing for synergistic effects. Future strategies may focus on developing multifunctional nanoplatforms that integrate imaging, targeting, and controlled therapy to overcome these limitations.

Cao et al. demonstrated a multifunctional theranostic platform combining porphyrin derivatives (P), known for strong singlet oxygen generation, with PEGylated graphene quantum dots (GQDs) functionalized with an aptamer (GQD-PEG-P) [176,181]. This agent exhibited excellent biocompatibility, physiological stability, and low cytotoxicity. The fluorescence of GQDs allowed tumor cell identification, while their surface facilitated intracellular microRNA detection. Notably, the platform achieved 28.58% photothermal conversion efficiency and a singlet oxygen quantum yield of 1.08, enabling effective PTT and PDT. Despite its high efficacy, challenges remain in translating such multifunctional systems to clinical applications, including reproducibility, scalability, and in vivo stability. Future research could focus on optimizing delivery, minimizing off-target effects, and enhancing patient-specific customization.

The combination of PDT and PTT offers several notable advantages, including synergistic therapeutic efficacy, the ability to activate systemic immune responses through immunogenic cell death, and dual functionality for imaging and therapy, which facilitates both diagnosis and treatment monitoring. However, there are significant limitations, such as restricted light penetration that limits effectiveness in deep-seated tumors, complex fabrication processes for multifunctional nanoplatforms, and challenges in optimizing dose and timing to achieve maximal synergistic effects. Future directions in this field include the development of more advanced and stable nanoplatforms, tailoring theranostic agents for patient-specific applications, and integrating these approaches with combinatorial treatments such as immunotherapy or chemotherapy to enhance overall treatment outcomes.

### 5.4. Red Light PDT

Cancer-targeting carbon quantum dots (CQDs) synthesized via a plasma electrochemical approach offer significant promise in biomedical applications, particularly photodynamic therapy (PDT). Conventional synthesis methods often require high temperatures and pressures, which can disrupt functional groups and diminish optical properties. In contrast, plasma electrochemical treatment preserves the molecular structure of precursors, maintaining both biological functionality and optical characteristics [161,182]. Wang et al. reported a rapid, environmentally friendly plasma electrochemical method to produce CQDs with red-light absorption (654 nm) and emission (660 nm), specifically targeting cancer cells. These FA-Ce6-Dots exhibit excellent water solubility, biocompatibility, and selective uptake by HeLa and HN6 cancer cells via folate receptor-mediated endocytosis, linked to pterins derived from folic acid [183]. Upon red-light excitation (638 nm), FA-Ce6-Dots efficiently generate reactive oxygen species (ROS), enabling effective tumor cell inactivation and demonstrating clear PDT potential [184].

Compared with non-targeted photosensitizers, which suffer from low selectivity and reduced efficacy, targeted CQDs offer a distinct advantage in precise ROS-mediated cytotoxicity. Singh et al. introduced a DNA-mediated assembly of ZnSe-CdS/ZnS quantum dots (QDs) conjugated with the photosensitizer protoporphyrin IX (PpIX) and a MUC1 aptamer, achieving specific recognition of the MUC1 cancer biomarker [185]. This system utilizes a multi-step fluorescence resonance energy transfer (FRET) mechanism, enabling both monitoring of aptamer binding and enhanced ROS generation upon irradiation. Circular dichroism and gel electrophoresis confirmed successful binding, while targeted photodamage was observed in MUC1-expressing HeLa cells.

Plasma electrochemical CQDs offer a simpler and environmentally friendly synthesis route that preserves optical functionality, making them suitable for scalable clinical applications. In contrast, DNA-mediated QD assemblies provide highly precise biomarker targeting through aptamer conjugation, but their preparation involves complex, multi-step procedures. Both approaches enable selective ROS generation for effective photodynamic therapy; however, CQDs are more straightforward and potentially easier to translate to in vivo applications, while DNA-mediated QDs provide modularity, allowing adaptation to a variety of cancer biomarkers. The advantages of CQDs include rapid and environmentally friendly synthesis, preservation of optical and biological functionality, water solubility, and biocompatibility, making them attractive for biomedical applications. DNA-QDs, on the other hand, offer high specificity through aptamer targeting, real-time FRET-based monitoring, and a modular design adaptable to various biomarkers. However, CQDs are primarily limited to cancers overexpressing folate receptors, and their in vivo biodistribution and long-term toxicity remain insufficiently explored. DNA-QDs face challenges related to complex and potentially costly synthesis, as well as stability under physiological conditions. Future directions involve expanding CQD targeting strategies beyond folate receptors to encompass other tumor-specific biomarkers, optimizing DNA-QD assemblies for improved stability and reduced synthesis complexity, and conducting comparative studies on therapeutic efficacy, biodistribution, and phototoxicity in animal models to guide clinical translation.

### 5.5. Chemo-(PTT:PDT)

Combination therapy integrating chemotherapy with near-infrared (NIR) light-mediated photothermal therapy (PTT) has emerged as a promising strategy for enhanced cancer treatment. NIR light offers deep tissue penetration and targeted activation, minimizing off-target damage. NIR-responsive nanomaterials can co-deliver chemotherapeutic and photothermal agents, generating localized heat upon irradiation. This heat increases tumor cell membrane permeability, improving drug uptake (e.g., doxorubicin, DOX) and enhancing cytotoxic effects [169,186]. Additionally, PTT can induce immunogenic cell death, triggering an anti-tumor immune response and potentially overcoming multi-drug resistance (MDR) through mechanisms like NIR-triggered nitric oxide release that downregulates P-glycoprotein [187].

While chemo–NIR combination therapy shows clear benefits in efficacy and reduced side effects [188], limitations include potential photothermal damage to surrounding tissues, heterogeneous tumor penetration, and complex nanomaterial synthesis. Comparatively, single-mode chemotherapy lacks spatial selectivity and often induces systemic toxicity, whereas standalone PTT may be insufficient to eliminate all cancer cells. Future directions involve optimizing NIR-responsive nanocarriers for precise spatiotemporal control, improving biocompatibility, and integrating imaging-guided theranostics for personalized treatment.

Zhang et al. demonstrated magneto-fluorescent carbon quantum dots (MCQDs) for chemo-phototherapy, synthesizing FeN@CQDs via a green hydrothermal method. Functionalization with riboflavin and folic acid (Rf-FA-FeN@CQDs) enabled light-triggered PTT and PDT, while embedding doxorubicin into polymer-integrated nanospheres (GP-Rf-FA-FeN@CQDs-DOX) facilitated targeted drug delivery. Confocal imaging confirmed cellular uptake, and in vitro/in vivo studies demonstrated synergistic tumor elimination under NIR irradiation [189,190]. This approach offers advantages of dual therapy and targeted delivery but requires careful control of particle size, surface chemistry, and potential long-term toxicity.

In a separate study, PEG-modified Fe_3_O_4_@CQDs encapsulated on single-walled carbon nanotubes (SWCNTs-PEG-Fe_3_O_4_@CQDs) were employed for imaging-guided therapy. Conjugation with sgc8c aptamer (SWCNTs-PEG-Fe_3_O_4_@CQDs-DOX-Apt) allowed dual fluorescence and MRI tracking. These nanocomposites exhibited combined photothermal and photodynamic effects under 808 nm laser irradiation and pH/NIR-triggered DOX release, demonstrating high efficacy against lung cancer cells [191]. Compared to the MCQDs system, SWCNT-based platforms offer enhanced imaging-guided precision and multi-modal therapy, though challenges remain in biodegradability, potential immunogenicity, and large-scale reproducibility. Future work should focus on improving targeting specificity, reducing off-target effects, and integrating stimuli-responsive, multifunctional nanoplatforms for clinical translation.

### 5.6. PDT-PTT

Photodynamic therapy (PDT) has long been explored for cancer treatment but is limited by low reactive oxygen species (ROS) generation, poor tissue penetration of conventional photosensitizers, and the short lifespan of ROS, all reducing therapeutic efficacy. To overcome these limitations, Zhang et al. developed a system combining rare-earth doped up-conversion nanoparticles (UCNPs) with graphene quantum dots (GQDs) [192]. UCNPs convert near-infrared (NIR) light into UV-visible emissions, which then excite GQDs to generate ROS efficiently. The addition of a mitochondria-targeting rhodamine derivative (TRITC) enhances intracellular accumulation, disrupts mitochondrial membrane potential, and induces apoptosis selectively in tumor cells. In vivo studies demonstrate superior tumor inhibition with organelle-specific UCNP-GQD/TRITC compared to non-targeting systems. While this approach significantly improves PDT efficiency, its reliance on complex nanoparticle synthesis may limit large-scale clinical translation. Future work could explore biodegradable or simpler alternatives to reduce potential toxicity.

Phototherapy, encompassing both PDT and photothermal therapy (PTT), offers versatile strategies by combining ROS generation and thermal effects. However, limitations exist: PTT requires prolonged high-energy laser exposure, and hypoxia or limited ROS diffusion can hinder PDT efficacy. Zhao et al. addressed these issues with multifunctional carbon dots (CDs) capable of simultaneously producing singlet oxygen, hydroxyl radicals, and heat under 635 nm laser irradiation [193]. These CDs demonstrate a quantum yield of 5.7% and a photothermal conversion efficiency of 73.5%, among the highest reported for CDs. Their lysosome-targeting enhances therapeutic specificity, while one- and two-photon fluorescence and photoacoustic imaging enable real-time theranostic monitoring. Comparison: UCNP-GQD systems focus on organelle-specific PDT, whereas CDs provide multifunctional PDT/PTT with dual imaging capabilities. High photothermal conversion may cause off-target tissue damage; optimization of laser parameters and biodistribution is necessary. Future research could explore tumor microenvironment-responsive CDs to further enhance safety and efficacy.

Targeting tumor-associated sialic acids has emerged as a promising strategy in cancer therapy due to their role in metastasis, immune evasion, and therapy resistance [194]. Silicon quantum dots (Si-QDs) functionalized with the photosensitizer Ce6 and phenylboronic acid (PBA) have been developed to exploit both PDT and PTT [195,196]. PBA conjugation improves cellular uptake and tumor accumulation, while light irradiation induces ROS generation and photothermal conversion, disrupting mitochondrial membrane potential and promoting apoptosis. The silicon quantum dots (Si-QDs) system offers the advantage of combining dual phototherapy with active targeting, resulting in synergistic tumor suppression. However, the complexity of surface engineering and the potential for off-target effects present notable challenges. To improve clinical applicability, future research could focus on developing biodegradable Si-QDs or incorporating stimuli-responsive linkers to enhance safety, efficacy, and translational potential.

Finally, Zhou et al. designed quantum dots conjugated with the RGD peptide for integrin-targeted PDT in pancreatic cancer cells [197]. The system effectively induced apoptosis, morphological changes, and cell cycle arrest while modulating key signaling proteins (Mcl-1, Akt, TRAIL). ROS production was confirmed, validating the therapeutic mechanism. Critical insight: This approach underscores the importance of targeting integrins to enhance PDT efficacy. A key limitation of this approach is that its success in vitro may not fully translate in vivo, owing to tumor heterogeneity and limited tissue penetration. To address these challenges, future studies should focus on integrating imaging-guided therapy and combination treatment strategies, which could enhance precision, improve therapeutic outcomes, and overcome the current barriers to clinical application.

### 5.7. PDT-CHT

**Figure 10:**
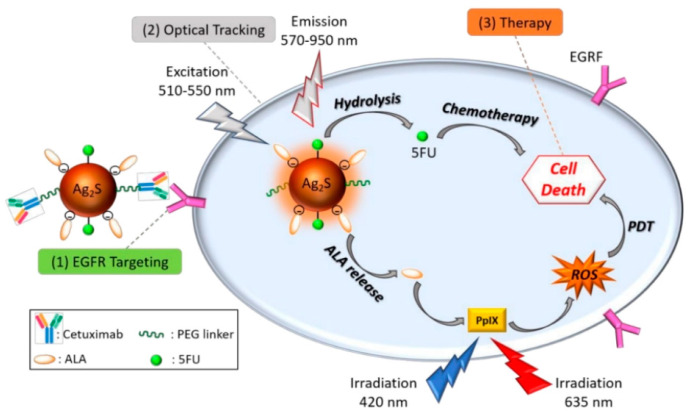
Theragnostic Nanoparticle Design for Targeted ALA-PDT Monotherapy and PDT/Chemotherapy Combination in EGFR-Positive CRC Cells. Reproduced with open access, license CC BY 4.0, from [199].

Colorectal cancer (CRC) has poor prognosis, highlighting the need for improved therapeutic strategies. Photodynamic therapy (PDT) with 5-aminolevulinic acid (ALA), which induces protoporphyrin IX (PpIX), is clinically established for several cancers, yet its use in CRC is limited due to poor tumor selectivity, particularly outside intracranial applications, restricting systemic adoption. Combining ALA-PDT with chemotherapy, such as 5-fluorouracil (5FU), may offer enhanced efficacy. Hashemkhani et al. proposed theranostic Ag2S quantum dots (AS–2MPA) conjugated with Cetuximab to target EGFR-positive CRC, loaded with ALA for PDT alone or combined therapy with 5FU [197,198]. This nanosystem demonstrates improved NIR detectability and targeted delivery, offering potential advantages over conventional ALA-PDT, including higher tumor specificity and theranostic capability. However, challenges remain, including potential quantum dot toxicity, complex synthesis, and limited in vivo validation. Future directions should focus on optimizing pharmacokinetics, minimizing off-target effects, and comparative studies with alternative nanoparticle carriers or targeted PDT strategies to establish clinical relevance Figure 10.

### 5.8. CDT-PDT

Carbon quantum dots (CQDs), known for their superior photoluminescence, are predominantly applied in bioimaging, with less exploration in targeted drug delivery [198]. To address this, a CQDs-based system, Arg-Ag@Cu, was designed using L-Arginine as a precursor and dual metal co-doping with silver (Ag) and copper (Cu) to improve doxorubicin (DOX) loading, release, and tumor theranostics [200]. Ag-doping enhances photoluminescence, while Cu^2+^ enables tunable CQDs size via interparticle self-assembly, increasing drug-loading capacity [201]. Functionally, the CQDs serve as NO/ONOO− donors for gas therapy, and Cu^2+^ contributes to reactive oxygen species (ROS) generation through chemodynamic therapy (CDT) and modifies the band structure for laser-induced photodynamic therapy (PDT), reinforcing ROS-mediated cytotoxicity [202]. The controlled depletion of Cu^2+^ triggers in situ DOX release, leveraging CQDs’ intrinsic bioactivity to inhibit tumor progression and migration while minimizing systemic side effects [203]. Compared to traditional CQDs or single-metal-doped systems, Arg-Ag@Cu offers synergistic multi-modal therapy with enhanced therapeutic efficacy, though challenges remain in precisely controlling metal doping ratios and in vivo pharmacokinetics.

### 5.9. Multimodal PDT-PTT, Photoacoustic

Biological systems exhibit high transparency to near-infrared (NIR) light (700–1100 nm), in which black phosphorus quantum dots (BP-QDs) demonstrate strong optical absorbance, integrating diagnostic and therapeutic functions in cancer theranostics [204]. In one study, BP-QDs were functionalized with targeting moieties (PEG-NH_2_-FA) and loaded with doxorubicin for combined photodynamic, photothermal, and chemotherapy. Folate conjugation facilitated selective internalization via folate receptors, ensuring targeted cytotoxicity upon NIR activation while sparing receptor-negative cells [205]. In vitro results confirmed effective photo-killing and precise, light-triggered drug release, and in vivo experiments demonstrated complete tumor elimination in mice without notable off-target toxicity. Additionally, the platform supports tumor growth inhibition and multimodal imaging, including photoacoustic and photothermal techniques [206]. While highly versatile, this nanoplatform’s design allows further optimization for enhanced therapeutic efficacy and multiplexed imaging, highlighting its potential for precision-targeted cancer treatment [207].

### 5.10. Sonodyanamic (SDT)

Developing effective sonosensitizers remains challenging due to the need to balance high sonosensitization efficacy with biocompatibility, which has limited the broader application of sonodynamic therapy (SDT). N-doped graphene quantum dots (N-GQDs) have emerged as promising sonosensitizers, combining the therapeutic advantages of SDT with the catalytic activity of graphene nanostructures. Notably, N-GQDs exhibit sonosensitization efficiencies 3–5 times higher than conventional sonosensitizers, including TiO_2_, porphyrins, and metalloporphyrins. Pyrrole N and pyridine N sites in N-GQDs likely serve as catalytic centers, enhancing sonochemical activity and offering insights into structure-dependent SDT optimization in carbon-based nanomaterials. Further functionalization with folic acid (FA-N-GQDs) enables selective tumor targeting, achieving over 96% marker affinity in tumor cells. In vitro and in vivo studies demonstrate tumor inhibition efficiencies exceeding 90%, mediated by oxidative stress via the PEX pathway and apoptosis through the p53 pathway [208].

N-GQDs offer several advantages, including high sonosensitization efficiency, excellent tumor selectivity, and favorable biocompatibility. However, potential limitations include off-target oxidative stress, limited long-term biosafety data, and challenges associated with large-scale production. Future directions involve exploring multi-functional doping strategies, combining SDT with other therapeutic modalities, and conducting detailed mechanistic studies to further enhance therapeutic efficacy while ensuring safety.

### 5.11. PDT-Imaging

Photodynamic therapy (PDT) is a non-invasive, photoactive cancer treatment whose efficiency depends on effective light exposure of the photosensitizer and selective accumulation in malignant cells. To address these challenges, folic acid and horseradish peroxidase (HRP)-functionalized semiconducting polymer dots (FH-Pdots) were developed as an integrated nanoplatform for targeted PDT and cancer cell imaging. In this system, meta-tetra(hydroxyphenyl)-chlorin (m-THPC) acts as a photosensitizer generating cytotoxic reactive oxygen species (ROS), while poly [2-methoxy-5-[[2-ethylhexyl)oxy)-p-phenylenevinylene] serves as a hydrophobic matrix and light-harvesting antenna. Surface modification with an amphiphilic Janus dendrimer allows conjugation of HRP and folic acid for selective targeting. The doped m-THPC can be excited via two pathways using the in-situ luminol–H_2_O_2_–HRP chemiluminescence system: directly through chemiluminescence resonance energy transfer (CRET) or via CRET followed by fluorescence resonance energy transfer [209].

In vitro studies on MCF-7 breast cancer cells, C6 glioma cells, and noncancerous NIH 3T3 fibroblasts demonstrated that FH-Pdots decreased cell viability in a dose-dependent manner, with greater cytotoxicity observed in cells expressing higher folate receptor levels. Fluorescence imaging confirmed higher uptake in cancerous cells compared to noncancerous controls, highlighting their targeting efficiency. Compared with conventional PDT, FH-Pdots offer the advantages of dual functionality—simultaneous imaging and therapy, enhanced targeting, and activation via chemiluminescence without external light sources. However, potential limitations include the complexity of synthesis, stability of functionalization, and limited in vivo validation. Future directions may involve optimizing biocompatibility, extending this approach to other cancer types, and integrating additional therapeutic modalities for synergistic effects.

### 5.12. CHT-PTT:PDT

**Figure 11:**
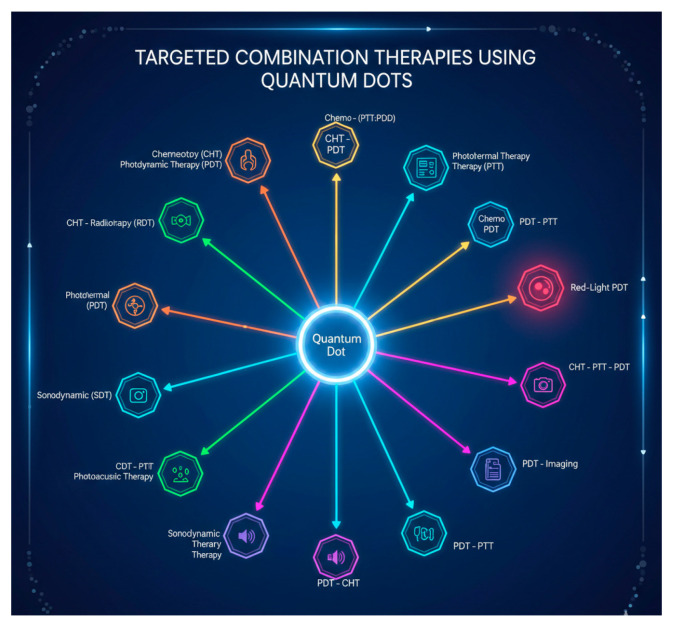
Illustration of some targeted combination therapies using quantum dots (Self-created).

The use of carbon-based quantum dots (CQDs) for drug delivery represents a promising advance in cancer therapeutics. Among these, carbon-dots clathrates (C-dotsCL) conjugated with folic acid (FA) enable controlled release of methotrexate (MTX) under physiological conditions, enhanced by photodynamic (PDT) and photothermal (PTT) therapy. The pH-sensitive C-dotsCL–MTX–FA complex demonstrates regulated drug release in vitro, while in vivo studies in genetically-induced pancreatic cancer models reveal preferential accumulation in tumor tissue compared to non-tumor tissue. Near-infrared (NIR, 1064 nm) laser irradiation further accelerates MTX release, and C-dotsCL efficiently generate reactive oxygen species (ROS), supporting combined therapeutic and imaging functionality. Pharmacokinetic improvements—including increased MTX half-life, reduced elimination, and higher AUC—highlight the system’s potential as an effective nanocarrier. Compared to conventional drug delivery, C-dotsCL offer targeted, multifunctional delivery with imaging capability, reduced systemic toxicity, and stimuli-responsive release. However, challenges remain, including potential long-term toxicity, scalability of synthesis, and precise control of in vivo biodistribution. Future research should explore surface modification for broader tumor targeting, combinatorial therapy strategies, and systematic safety evaluations to translate this platform into clinical use [210]. Novel conjugated carbon dots (CDs) were developed as two-photon active photosensitizers for precise nucleus-targeted therapy. Folic acid and curcumin conjugation enhanced nuclear internalization and minimized non-specific uptake, enabling near-infrared (NIR)-triggered ROS generation. This strategy directly induced cancer cell apoptosis by targeting DNA, demonstrating a multifunctional platform for enhanced photodynamic therapy (PDT) in oral cancer theranostics [211], Figure 11.

## 6. Quantum Dots in Targeted Imaging and Theranostic

### 6.1. Targeted Imaging and Therapy

**Figure 12:**
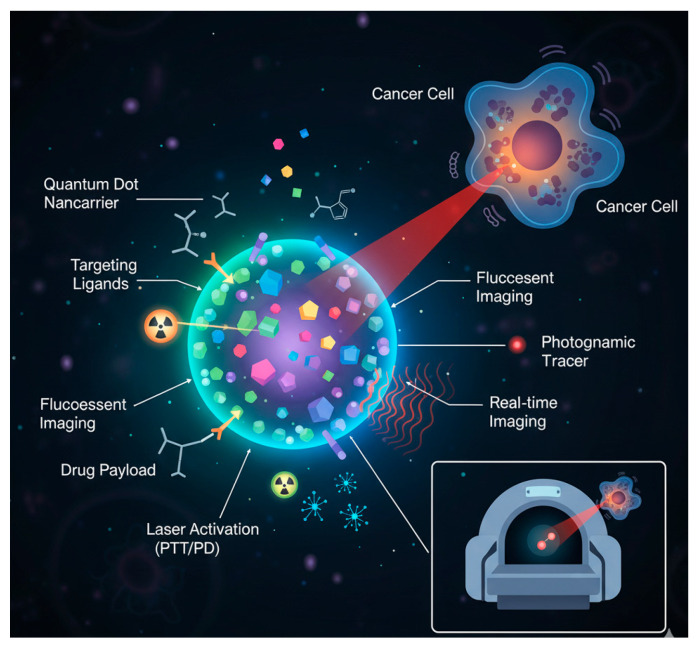
Illustration of quantum dots in imaging and theranostics (Self-created).

Developing safe and effective nanoprobes for targeted imaging and selective therapy of in-situ gastric cancer remains a significant challenge. In this context, a versatile HER2 monoclonal antibody-conjugated RNase A-associated CdTe quantum dot cluster (HER2-RQDs) nanoprobe was synthesized, and its cytotoxicity was thoroughly examined, Figure 12. Both in-situ gastric cancer SCID mouse models and subcutaneous gastric cancer nude mouse models were employed to evaluate the efficacy, where HER2-RQDs nanoprobes were intravenously administered, and their biodistribution and therapeutic effects were quantified [212,213].

The results indicated that HER2-RQDs nanoprobes preferentially targeted and killed gastric cancer MGC803 cells, enabling imaging of subcutaneous tumors within 3 h post-injection and in-situ gastric tumors by 6 h post-injection. The nanoprobes also inhibited tumor progression and prolonged the lifespan of gastric cancer-bearing mice, primarily through RNase A-mediated degradation of functional cytoplasmic RNAs, leading to inhibition of protein synthesis and activation of apoptosis.

**Figure 13:**
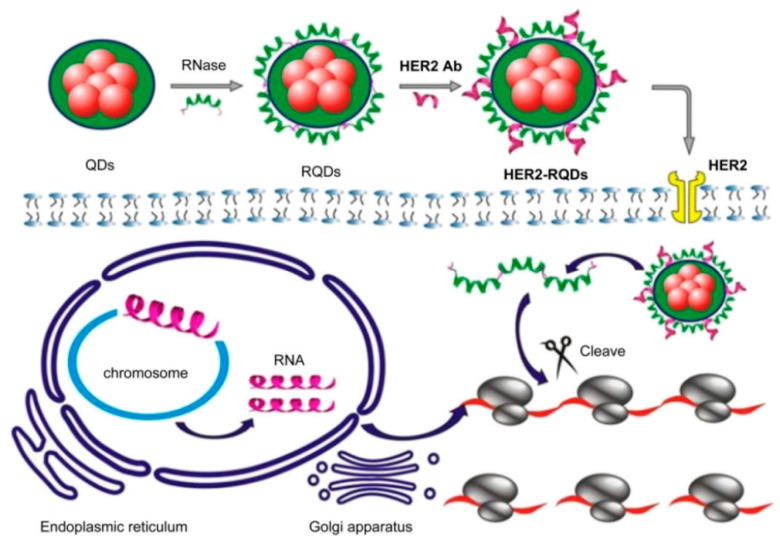
Therapeutic mechanism of synthesized nanoprobes. Reprinted with permission from: Elsevier [36].

Compared with conventional chemotherapeutic approaches, HER2-RQDs nanoprobes demonstrate significant advantages by offering dual functionality, enabling both targeted imaging and selective cytotoxicity, thereby minimizing off-target effects. Traditional systemic chemotherapy, in contrast, lacks such spatiotemporal specificity and often induces considerable toxicity in healthy tissues. Other nanoparticle-based systems, including liposomal or polymeric carriers, provide controlled drug release but generally exhibit lower targeting specificity and slower tumor uptake compared to HER2-RQDs [214]. The main advantages of HER2-RQDs include their high specificity for HER2-positive gastric cancer cells, the ability to perform real-time imaging alongside therapy, and reduced systemic toxicity relative to conventional treatments. However, potential limitations exist, such as the risk of immunogenicity or long-term accumulation of CdTe quantum dots in vivo, limited penetration within dense tumor microenvironments which may reduce efficacy in larger or hypoxic tumors, and variability in RNase A activity depending on intracellular conditions, which can affect reproducibility. Future research should focus on integrating biodegradable or non-toxic quantum dot alternatives to mitigate heavy-metal-related risks, combining HER2-RQDs with complementary therapeutic modalities like photothermal therapy to enhance efficacy, and optimizing nanoprobe pharmacokinetics and tumor penetration to improve treatment outcomes in heterogeneous gastric tumors. Overall, HER2-RQDs nanoprobes represent a promising platform for in-situ gastric cancer-targeted imaging and selective therapy, with potential for further clinical translation upon addressing the aforementioned limitations, Figure 13 [36,215].

### 6.2. Imaging with PTT

Carbon quantum dots (CQDs) have emerged as versatile agents for optical bioimaging due to their fluorescence properties. However, challenges remain, including high-cost precursors, low quantum yield, and complex fabrication methods, as well as limited selectivity between cancerous and normal cells. To overcome these issues, a single-step, cost-effective hydrothermal method was developed using folic acid and citric acid to produce water-dispersible, high quantum yield CQDs. For targeted imaging, a biocompatible, non-toxic nanoparticle-antibody conjugate radiolabeled with 99mTc, incorporating GQDs and pembrolizumab, enabled SPECT imaging of breast cancer in 4T1 tumor-bearing BALB/c mice, demonstrating effective tumor targeting and favorable pharmacokinetics [216].

Polypyrrole (PPy) nanoparticles are also widely used for photothermal therapy (PTT) owing to their strong NIR absorption. Yet, their therapeutic efficiency is constrained by limited targeting precision. To address this, PPy nanoparticles were combined with nitrogen-doped CQDs (NCQDs) and folic acid to form PPy:NCQDs:FA nanocomposites. These composites exhibited strong photothermal properties, green fluorescence imaging of folate receptor-positive MCF-7 cells, and high biocompatibility, maintaining over 82% cell viability at 600 μg mL^−1^ [215].

MXene-based FHMQDs (~3 nm) represent another advancement in theranostic design. These quantum dots were engineered for stimuli-responsive behavior, breast cancer cell selectivity, and simultaneous drug delivery and imaging. FHMQDs efficiently encapsulated doxorubicin (~90% loading) and released it preferentially at acidic pH (5.4). In vitro studies showed strong cytotoxicity toward MDA-MB-231 cancer cells, ROS-mediated photodynamic effects, apoptosis induction, and fluorescent labeling for bioimaging, highlighting their potential as integrated theranostic platforms [217].

Comparatively, carbon quantum dots (CQDs) offer the advantages of simplicity, cost-effectiveness, and ease of synthesis, making them attractive for bioimaging applications; however, they face challenges in long-term stability and in vivo specificity, which can limit their therapeutic precision. Polypyrrole-based NCQD-folic acid (PPy:NCQDs:FA) nanocomposites improve targeting and imaging accuracy due to folate receptor-mediated uptake and strong photothermal properties, but their performance depends on careful optimization of the fluorescence–photothermal balance to minimize off-target effects and ensure biocompatibility. Titanium carbide-based MXene quantum dots (FHMQDs) provide a multifunctional theranostic platform that integrates drug delivery, imaging, and combined photothermal/photodynamic therapy, yet their complex synthesis and potential immunogenicity present hurdles for widespread application. Looking forward, future efforts should focus on developing multifunctional CQDs or MXene QDs capable of dual or multi-modal imaging and therapy with enhanced biocompatibility, refining tumor-specific targeting through ligand modification or stimuli-responsive mechanisms, and systematically evaluating long-term in vivo toxicity, clearance pathways, and translational feasibility to advance these nanomaterials toward clinical applications.

### 6.3. QDs-Hydrogels, Nanocomposites, and Layer-by-Layer System

Mg/N-doped carbon quantum dots (CQDs) with dual drug-targeting and cell-imaging capabilities were synthesized via a hydrothermal method, optimizing pyrolysis parameters such as pH, temperature, and duration to enhance quantum yield. Functionalized with hyaluronic acid and folic acid (CQD-FA-HA) and loaded with epirubicin (CQD-FA-HA-EPI), these nanoprobes demonstrated cytotoxicity, cellular uptake, and imaging efficiency in MCF-7, 4T1, and CHO cell lines, and in vivo efficacy in BALB/c mice [218].

Targeting QDs on nanofibers offers enhanced specificity and efficacy due to structural advantages and controlled drug release; however, biocompatibility and long-term safety require further validation [219].

The layer-by-layer (LbL) electrostatic assembly enables nanoscale film formation using oppositely charged polymers, such as proteins and polysaccharides, which can target tumors efficiently [220]. Gelatin/chondroitin nanocapsules co-encapsulating rapamycin (RAP) and celecoxib (CXB) demonstrated enzyme-responsive, MMP-2-degradable cationic coatings, enabling selective uptake in breast cancer cells with minimal off-target effects. In vivo studies confirmed enhanced anticancer efficacy and low immunogenicity [221] (Figure 13).

**Figure 14:**
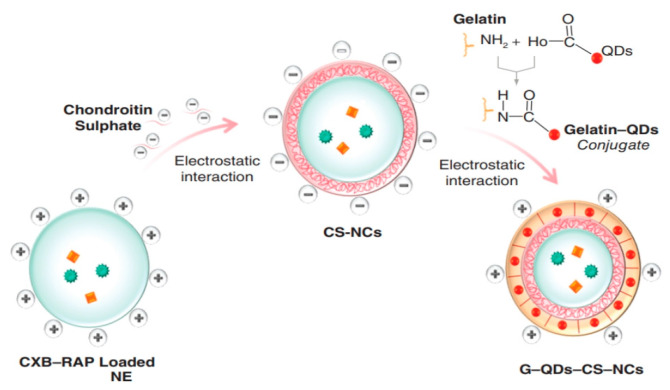
A schematic diagram depicting the stepwise preparation process of layer-by-layer gelatin/chondroitin quantum dots-based nanotheranostic system. (CXB: Celecoxib; G: Gelatin; NE: Nanoemulsion; QD: Quantum dot; RAP: Rapamycin). Reproduced with permission from [223].

Similarly, theranostic lactoferrin (LF)/chitosan (CS) LbL QD nanohybrids co-delivering honokiol (HNK) and CXB achieved dual targeting via CD44-mediated endocytosis and LF receptor interactions, Figure 14. CdTe QDs provided imaging-guided drug release, with luminescence switching from OFF to ON upon cellular internalization. In vitro and in vivo studies showed superior antitumor effects compared to free drugs [199,222,223].

Quantum Dot DNA Hydrogels (QDHs) offer notable advantages over conventional [214] drug delivery systems: biocompatibility, reduced cytotoxicity, targeted ligand-mediated delivery, enzyme-responsive/stimuli-triggered release, multifunctionality (drug delivery, imaging, biosensing), and simplified self-assembly synthesis [192,224,225,226,227,228]. Conventional carriers often exhibit systemic toxicity, limited specificity, and single-functionality, highlighting the superiority of QDHs. Limitations include potential scale-up challenges and incomplete understanding of long-term in vivo stability. Future directions include exploring diverse DNA motifs for precise stimuli-responsiveness and multifunctional theranostics.

Polymeric micelles (PHEA-LA-PEG-FA) and nanostructured lipid carriers (NLCs) co-encapsulating QDs and paclitaxel demonstrated tunable size, zeta potential, high drug loading, and controlled biphasic release, achieving high cytotoxicity and targeted tumor imaging [229,230]. Comparatively, hybrid QD–immunoliposome (QD-IL) systems targeting EGF receptors via ligand-directed bulk-flow delivery enhanced glioblastoma uptake and imaging precision over non-targeted formulations [231]. Limitations include potential immunogenicity and complex manufacturing processes, suggesting further optimization for clinical translation.

Hybrid hydrogel carriers integrating doxorubicin (DOX)-loaded PEG microspheres with peptide-carbon dot nanowires showed synergistic anti-cancer effects and specific targeting of MDA-MB-231 breast cancer cells, with improved biodegradability and controlled release [232,233,234].

DNA-templated QDHs further allow tunable optical properties, nine-fold enhanced DOX delivery, and multifunctionality, demonstrating promise for traceable, enzyme-responsive, and clinically relevant theranostics [35]. Limitations include potential immunogenic responses and the need for extensive in vivo validation. Future research should explore long-term safety, scalability, and integration with combinatorial therapies.

QDs integrated into hydrogels, LbL systems, and nanocomposites offer enhanced targeting, multifunctionality, and controlled release compared to conventional drug carriers. Pros include imaging-guided therapy, stimuli-responsive delivery, and improved cytotoxicity; cons involve complexity in synthesis, potential immunogenicity, and scale-up challenges. Future directions emphasize optimizing biocompatibility, expanding multifunctional platforms, and enabling clinical translation.

## 7. Conclusions and Future Outlook

Quantum dots (QDs) are semiconductor nanocrystals with unique size-dependent electronic and optical properties arising from quantum confinement effects. These properties allow tunable light emission and have made QDs highly versatile in applications ranging from electronics and photonics to biomedical technologies. In medicine, QDs have demonstrated significant promise for high-resolution fluorescence imaging, targeted drug delivery, and photothermal therapy. Functionalization with targeting ligands enhances site-specific delivery of therapeutics, potentially reducing off-target effects. Additionally, their intrinsic photothermal properties offer innovative strategies for cancer treatment, such as localized tumor ablation [2,7].

Despite these advances, several gaps and challenges remain. Toxicity and biocompatibility continue to limit the clinical translation of traditional cadmium-based QDs, and while cadmium-free alternatives are emerging, their long-term safety, stability, and efficacy are not yet fully established. Conflicting results in the literature regarding biodistribution, clearance, and cellular uptake of QDs highlight the need for standardized protocols and more comprehensive in vivo studies. Moreover, scalability and reproducibility in QD synthesis pose technical challenges, particularly for biomedical applications where precise control over size, surface chemistry, and optical properties is critical.

From a commercial standpoint, the QD market is poised for substantial growth, projected to rise from approximately $6 billion in 2024 to over $25 billion by 2032, driven by demand in display technologies, biosensing, and medical imaging [1,3,5,18]. However, while applications in electronics are relatively mature, biomedical applications still face regulatory, safety, and manufacturing hurdles.

Future directions should focus on:

1. Safer and sustainable QD formulations: Development of non-toxic, environmentally compliant QDs with high quantum yield and stability.
2. Standardization of biological studies: Establishing uniform protocols for assessing in vivo pharmacokinetics, toxicity, and therapeutic efficacy.
3. Integration with emerging technologies: Combining QDs with CRISPR, AI-based imaging, and nanotheranostics to enhance personalized medicine.
4. Addressing conflicting results: Systematic comparative studies across different QD types, sizes, and functionalizations to resolve inconsistencies in biodistribution, clearance, and cellular interactions.
5. Scalable manufacturing: Advancing reproducible and cost-effective synthesis methods to facilitate commercialization for both biomedical and electronic applications.

In conclusion, quantum dots represent a rapidly evolving field with transformative potential across multiple sectors. Addressing current knowledge gaps, resolving conflicting findings, and prioritizing safety and sustainability will be essential for translating QD research into clinically and commercially viable technologies. With continued innovation and strategic integration, QDs are likely to play a pivotal role in next-generation diagnostics, therapeutics, and high-performance electronic devices.

## Figures and Tables

**Table 2:** Comparison of the characteristics and applications between traditional organic fluorophores and QDs.

Property	Traditional Organic Fluorophores	Quantum Dots (QDs)	References
Chemical properties	Poor chemical resistance	Chemically resilient; pH sensitivity determined by surface coatings	[43,53,54,55,56,57,58,59,60]
Dimensions	Molecular (<0.5 nm)	Colloidal (1.5–10 nm diameter)	[43,53,54]
Hydrodynamic radius	<0.6 nm ^a^	1.4–40 nm ^b^ (depends on coating and ligand)	[61,62]
Absorption spectra	Discrete bands, FWHM ≈ 35–100 nm ^cde^	Strong and broad absorption	[56,57]
Emission spectra	Broad, red-tailed, asymmetric, FWHM ≈ 35–100 nm	Narrow, symmetric, FWHM ≈ 30–90 nm	[56,57,61,62]
Two-photon cross-section	10–500 GM	2000–47,700 GM ^f^	[56,58]
Molar absorption coefficient	10^3^–10^5^ cm^−1^ mol^−1^ L	10^5^–10^6^ cm^−1^ mol^−1^ L	[43,53,54,55,57,58,59,60]
Quantum yield	0.05–1.0	>20% ^g^ (ligand/shell dependent)	[57,58,59,60]
Fluorescence lifetime	<5 ns, mono-exponential	>10 ns, multi-exponential	[55,61]
Solubility/Dispersibility	Determined by substitution pattern	Controlled via surface chemistry (ligands)	[53,56]
Thermal stability	Variable; depends on dye	High; shell/ligand dependent	[57,62]
Photostability	Poor; prone to photobleaching	Excellent; long observation time	[55,56,57]
Bioconjugation labels	Mostly monovalent	Multivalent scaffolds; diverse conjugation	[58,59]
Single-molecule analysis	Limited by bleaching	Effective; restricted by blinking	[57,61]
Spectral multiplexing	Possible but limited	Excellent; ≥5 distinct colors achievable	[56,57,58]
Multifunctionality	Difficult and rare	High potential for multifunctional integration	[57,58,59,60]
Toxicity	Depends on dye chemistry	Related to heavy-metal content (e.g., Cd, Pb)	[55,57,62]

^a^: Except for fluorescent proteins, GFP 4.6 × 2.4 nm cylindrical shape ^b^: Coating, ligand, and bioconjugate-dependent ^c^: FWHM, full width at half height of the maximum. ^d^: Dyes with resonant emission, such as fluorescein, rhodamine and cyanine. ^e^: CT dyes. ^f^: Wavelength-dependent; GM: Goeppert–Mayer units ^g^: Ligand, coating and solvent-dependent.

**Table 3:** Various methods employed to fabricate QDs.

Methods of Fabrication	Quantum Dots Engineered	Characteristics	Refs.
Electron beam lithography	QD nanostructures	Optical properties preserved after cross-linking	[66]
	QD microarrays	Fluorescence Bioaffinity	[67]
Reactive ion etching	Indium gallium nitride (InGaN) QDs	Strong and distinct photoluminescence signal	[68]
Sol-gel	Titanium dioxide (TiO_2_) QDs	Extensive surface area, photocatalytic properties	[47]
	Zinc selenide (ZnSe) QDs encapsulated in Silicon dioxide (SiO_2_)	-	[48]
	Cadmium sulfide (CdS) and Ni-doped CdS	Highly crystalline	[38]
	Zinc oxide (ZnO)@polymer core/shell	Quantum yield above 50%	[39]
	Zinc oxide (ZnO) QDs	Strong photoluminescence efficiency	[30]
Microemulsion (reverse micelle)	Zinc sulfide (ZnS) QDs	Nanocrystal with high purity, Photoluminescence peak observed at 365 nm Quantum confinement effect	[51]
	Cadmium sulfide/Zinc sulfide (CdS/ZnS) semiconductor QDs	Excellent luminescence and photostability	[51]
	Cadmium selenide@Zinc sulfide (CdSe@ZnS) within monodisperse silica	Good monodispersity High luminescence	[63]
Microemulsion (gas contacting technique)	Zinc selenide (ZnSe) QDs	Excellent photostability and size-influenced luminescence	[69]
Microemulsion method + ultrasonic waves (sono-microemulsion method)	Cadmium sulfide (CdS)	Restricted size distribution High-order crystalline arrangement and purity	[70]
Physical vapor deposition	Niobium pentoxide (Nb_2_O_5_) QDs	Quantum confinement effect	[21]
RF magnetron sputtering	Cadmium selenide (CdSe) QDs	Optical properties	[69]
Solvothermal	Zinc Oxide (ZO) QDs	Minuscule size High purity, superior crystallinity, and large surface area	[70]
	Graphene QDs (GQDs)	Resilient stability, photoluminescence quantum yield of 11.4%, biocompatibility, mild toxicity	[21]
Hydrothermal	Nitrogen- and sulfur-doped carbon QDs (N, S-doped CQDs)	Small Spherical Green emission	[20]
		Fluorescence quantum yield (10.35%)	
	Nitrogen-doped carbon QDs (N-CQDs)	Low toxicity excellent photostability	[40]
	Silicon QDs	Excellent water dispersibility High photoluminescence Strong pH stability	[71]
	Tin oxide/Tin sulfide in reduced bovine serum albumin (SnO_2_/SnS_2_ @r-BSA2)	Specific selectivity Long term stability Enhanced reproducibility	[72]
	Nitrogen-doped Graphene QDs (N-GQDs)	High quanta yield Persistent fluorescence stability Enhanced sensitivity and specificity	[73,74]
Molecular beam epitaxy	Indium arsenide gallium arsenide core/shell (InAs/GaAs) QDs	Strong photoluminescence intensity High structural properties	[75]

**Table 4:** Dimension of quantum dots and corresponding emission spectra Reprinted with permission from [105].

Quantum Dot of Compounds	Size Spectrum (Diameter in nm)	Range of Emission Spectrum (nm)
**Cadmium sulfide (CdS)**	2.8–5.4	410–460
**Cadmium telluride (CdTe)**	3.1–9.1	520–750
**Cadmium selenide (CdSe)**	2–8	480–680
**CdTe/CdSe**	4–9.2	650–840
**Indium phosphide (InP)**	2.5–4.5	610–710
**Indium arsenide (InAs)**	3.2–6	860–1270
**Lead selenide (PbSe)**	3.2–4.1	1110–1310
**1-Dodecanethiol silver sulfide (Dt)-Ag_2_S)**	5.4–10	1000–1300

**Table 5:** An overview of surface functionalization methods for quantum dots (QDs), (highlighting the benefits and limitations of the four primary techniques).

Surface Optimization Techniques	Advantages	Disadvantages	Refs.
**Ligand exchange**	Feasibility of processing, Small dimensions of QD	Degradation of photophysical properties in QDs present in aqueous environment (i.e., reduced PLQY) QD core is suspected to be oxidation	[94,110,111,112]
**Surface silanization**	Enhances biocompatibility, High cross-linking in ligand molecules, Terminal groups enable further coating by exposing their reactive ends (e.g., thiol), Fine-tuning the QD response to light is enabled by controlling the thickness of the silica shell, Improves PLQY of QDs, Improves photochemical stability.	Large hydrodynamic size, Aggregation of QDs in aqueous solution	[113,114,115]
**Amphiphilic ligands**	Increased chemical stability, Increased colloidal stability, Excellent biocompatibility, and strong fluorescence signals with high stability.	Size enlargement, Surface defects	[112,113,116]
**Microsphere coating**	Improvement in the stability of QD, High fluorescence, Effectively conceals QD toxicity	The formation of a uniform microsphere is obstructed, Reduced PLQY, Encapsulating the QDs with high concentrations finds QD aggregation.	[114,117]

## Data Availability

No new data were created or analyzed in this study.
